# Transcriptional footprints associated with bud fertility in grapevine development (*Vitis vinifera* L.)

**DOI:** 10.1007/s00425-026-05000-3

**Published:** 2026-04-08

**Authors:** Francesco Girardi, Giorgia Bettio, Angela Rasori, Monica Canton, Alessandro Botton

**Affiliations:** 1 Department of Agronomy, Food, Natural resources, Animals and Environment - DAFNAE, University of Padova, Agripolis, - Viale dell’Università 16, 35020 Legnaro - PD, Italy; 2 Interdepartmental Research Centre for Viticulture and Enology - CIRVE, University of Padova, Via XXVIII Aprile 14, 31015 Conegliano, Treviso, Italy

**Keywords:** Bud fruitfulness, Hormones, RNA-Seq, Phenological stage, Node position, Floral transition

## Abstract

**Main conclusion:**

Transcriptomic analyses carried out on buds at different positions and developmental stages reveal the functional domains mainly involved in determining bud fertility in grapevine, with a focus on hormone signalling and floral integrators.

**Abstract:**

Bud fruitfulness in grapevine (*Vitis vinifera* L.) depends on complex interactions between developmental stage, node position and hormonal regulation. In this study, transcriptomic analyses were performed on buds of cv. Merlot collected at three phenological stages (BBCH63, BBCH77 and BBCH90) from three node positions (2, 5 and 10), representing basal, intermediate and apical fertility patterns. RNA-Seq data revealed that phenological stage was the primary driver of transcriptional variation, while node position contributed to intra-stage differences. BBCH77 emerged as the most transcriptionally dynamic phase. Differential expression, enrichment analyses and WGCNA highlighted distinct developmental trajectories among nodes. Buds at node 5, characterized by lower fertility, showed transcriptional signatures associated with enhanced gibberellin biosynthesis and signalling, early activation of dormancy-related pathways, and complex hormonal interplay involving ethylene. In contrast, buds at node 10 displayed transcriptional features consistent with sustained auxin flux from the shoot apical meristem and reduced local inhibitory signalling, potentially supporting higher fertility. Cytokinin-related genes showed stage-dependent activation, particularly at BBCH77, suggesting a transient promotive role during floral transition. Expression profiling of flowering-related genes confirmed stage-dependent regulation of major floral integrators and revealed position-specific modulation of key regulators. Overall, the integration of positional and temporal transcriptomics supports a working model in which bud fertility results from the dynamic balance between stimulatory (auxin, cytokinins) and inhibitory (gibberellins, ethylene, ABA) signals, modulated by developmental stage and meristem proximity.

**Supplementary Information:**

The online version contains supplementary material available at 10.1007/s00425-026-05000-3.

## Introduction

Flowering in grapevine is a pivotal developmental process that influences both the quantity and the quality of grape production. As a general concept, this process involves the transition of the meristem from vegetative to reproductive growth, culminating in the formation of flowers and, next, to anthesis (Raghavan 2000). This process is intricately regulated by both environmental cues and internal genetic programs, ensuring that flowering occurs at the optimal time for successful pollination and fruit set.

Grapevine buds develop over two growing seasons separated by a period of dormancy, making their study particularly challenging and intriguing (Vasconcelos et al. 2009). During the first season, the shoot apical meristem within the bud at the leaf axil differentiates and forms the basic elements of the shoot for the following year, including the initiation of the inflorescence primordia, as a response to both exogenous (i.e., high temperatures and high light radiation) and endogenous factors. This phase ends with the onset of bud dormancy, a state in which growth is temporarily halted to survive the unfavourable environmental conditions of winter (Díaz-Riquelme et al. 2012). In the subsequent season, buds resume growth, leading to completion of flower development and, finally, to anthesis.

The development of grapevine buds can be divided into distinct stages: induction, transition, initiation, differentiation, dormancy, reactivation, and anthesis (Pellegrino et al. 2020; Monteiro et al. 2021). During the induction phase, the buds collect a series of environmental and endogenous signals, which stimulate the meristem to change its identity and transit from the vegetative to the reproductive phase (floral transition). Once transited, the buds undergo the initiation stage, and the lateral meristems develop into uncommitted primordia, which can later differentiate into either tendrils or inflorescences (Boss et al. 2003). Differentiation involves the formation of inflorescence primordia, which later develop into fully formed inflorescences.

Physiological and molecular events underpinning bud development in grapevine are complex and involve numerous genes and signalling pathways. The transition from vegetative to reproductive growth is regulated by key floral integrator genes, such as *VvFT* and *VvMADS8/VvSOC1*, which are homologous to the *Arabidopsis FLOWERING LOCUS T* (*FT*) and *SUPPRESSOR OF OVEREXPRESSION OF CONSTANS1* (*SOC1*) genes (Boss et al. 2006). Besides being expressed in leaves at floral induction (Crane et al. 2012), *VvFT* transcripts have also been found in developing inflorescences, ovules, stamens, and berries, suggesting a multiple role for this gene in promoting flowering and fruit/seed development (Sreekantan and Thomas 2006). Conversely, *VvMADS8* (also known as *VvSOC1*) is expressed primarily in axillary buds during the initial phases of flower transition/initiation (Sreekantan and Thomas 2006; Almada et al. 2009; Díaz-Riquelme et al. 2014). *TFL1A* expression was shown to be fundamental to achieve optimal fertility in grapevine, although this gene is generally considered a floral repressor. It is the ortholog of Arabidopsis *TFL* and exhibits a conserved function when overexpressed in both Arabidopsis and tobacco, where it induces an indeterminate growth pattern of the meristem (Boss et al. 2006; Carmona et al. 2007). In grapevine, *TFL1A* is expressed in the SAM (shoot apical meristem) to prevent it from developing a terminal flower that would otherwise close shoot development. Therefore, its prolonged expression in the buds allows the inflorescence primordia to undergo a higher level of branching before transiting to the reproductive phase (Boss et al. 2006). Indeed, experiments carried out by Crane et al. (2012) attempted to correlate the expression patterns of both *VvFT* and *VvTFL1A* with the fertility gradient assessed in buds at different nodes, pointing out a perfect synchronism and relative dosages of the expression of both genes. The FT signal wave, coming mainly from leaves through the phloem, must not arrive to the bud too early, to allow the primordia to reach a sufficient level of branching thanks to *TFL1A* (and *VvVFL*) expression. That is what happens in more fertile apical buds, while in less fertile basal buds, their more advanced stage of development triggers the expression of *VvFT* too early, when the inflorescence primordia have not yet reached a sufficient branching level. A similar situation has also been demonstrated in Arabidopsis, where the control of *TFL1* is pivotal to flexibly counteract incoming FT signals, allowing a pool of undifferentiated cells to be maintained despite differentiation signals spreading in nearby cells (Jaeger et al. 2013).

Together with these fundamental genetic determinants of flowering, hormones are deeply involved in the process. One of the pillars of knowledge about the involvement of hormones in grapevine flowering is provided by the pioneering work carried out on Pinot Meunier grapes by Boss and Thomas (2002), who unequivocally demonstrated the negative effect of gibberellins (GAs) on floral induction. Oppositely to GAs, the cytokinins were demonstrated to exert a positive regulation on flowering, even with the ability to transform the tip of a young tendril into an inflorescence determining consistent effects also on the flowering genes (Crane et al. 2012).

Besides these endogenous factors and genetically programmed mechanisms, environmental factors such as photoperiod, light intensity, and temperature profoundly influence the timing of bud development and flowering. The interaction between environmental signals and endogenous florigenic stimuli ensures that flowering occurs at the optimal time, maximizing reproductive success. For example, the *CONSTANS* (*CO*) gene and its downstream targets, such as *FT*, integrate photoperiodic signals to regulate flowering time. In grapevine, *VvCO* activates the expression of FT, which then moves to the shoot apex to initiate flowering, despite European grapevine is a facultative photoperiodic species (Sreekantan and Thomas 2006; Noyce 2017).

Unfortunately, the information provided above is fragmentary and derives from studies carried out over several decades, in both hemispheres, different genotypes and under different environmental conditions. Therefore, a univocal model cannot be built unless all this information is validated within a single experiment. The present study aims to support the construction of a new and updated working model for grapevine flowering by means of a transcriptomic approach. Buds were collected from different nodes of the growing shoot over different phenological phases that included floral induction, transition, and initiation. Based on the fertility of the different nodes assessed in the following year, the most representative samples were chosen, their transcriptomes sequenced and the gene expression investigated to: i) validate previous studies to start from a robust knowledge base, ii) enhance the information about the gene functions involved in the flowering process, iii) identify genes able to mark buds at specific positions and phenological stages, and iv) build a working model of the transcriptional footprints associated with bud fruitfulness in grapevine.

## Materials and methods

### Experimental design and plant material

The main experiment was carried out in 2023/2024 at ‘La Mincana’ farm, located in Due Carrare (Padova, Italy; 45°18′02.9"N 11°47′48.3"E), on 209-year-old grapevines cv. Merlot grafted onto Kober 5BB rootstock. The vines were trained to a spur-pruned cordon system and normally pruned with two buds. Lateral shoots were pruned every 10 days to keep the main shoot dominant throughout the whole season. Agronomic practices conducted in the vineyard included standard viticultural operations for wine grape production, such as phytosanitary treatments and canopy management. No soil management or fertilization was implemented. Five key sampling sessions were established throughout the 2023 growing season. The sampling dates and corresponding phenological stages (Lorenz et al. 1995) were as follows: June 16th, BBCH63 (anthesis); June 26th, BBCH71-73 (fruit set and berry development); July 3rd, BBCH73-75 (berry development); July 10th, BBCH77 (cluster closure); and October 10th, BBCH90 (harvest). During each session, approximately thirty shoots of similar size were randomly collected from different positions along the cordon and different vines of the row, excluding unhealthy plants. The shoots were immediately placed in water to maintain hydration, then buds were promptly collected from nodes 1 (Bourillon), 2, 3, 4, 5, and 10 (Supplementary Fig. 1A). Buds excised from three biological replicates, each constituted by a pool of buds of the same node, were immediately placed in liquid nitrogen and then stored at -80 °C for the following analyses.

### Fertility measurements

For the main transcriptomic experiment, samples were collected in 2023 and fertility was assessed in 2024 on fifty vines of the same vineyard that were kept pruned to ten buds, to be able to study the fertility of the first ten nodes that were previously sampled. At BBCH57 phenological stage (‘*inflorescences fully developed; flowers separating’*; Lorenz et al. 1995), all inflorescences were removed from each shoot at each node separately, counted and weighed individually (Supplementary Fig. 1B). Since at BBCH57 the phenology of the different shoots was very synchronous, thanks also to the careful management of the vineyard, these measurements were carried out in a single date. In addition, fertility was assessed in three consecutive years (2022, 2023, and 2024) also on fifty vines of the same cv/rootstock grown at the Experimental Farm of the University of Padova, to assess reliability in the fertility pattern of cv. Merlot in a different location.

### RNA extraction, RNA-Seq analysis and qPCR

Total RNA was extracted with the RNeasy Plant Mini Kit (Qiagen) starting from 70 mg of tissue, with the following modifications made to the protocol provided by the manufacturer: 5 $\mu$l of Ca(OH)_2_ and 0.05 g of PVP were added to 750 $\mu$l of the Extraction Buffer RLT. The former was added for its ability to precipitate carbohydrates according to Dal Cin et al. (2005), while PVP was added to precipitate polyphenolic compounds. The amount of RNA and the presence of contaminants in the extracted samples were evaluated using NANO drop 2000 (EuroClone^®^), and RNA integrity was checked by running the samples in a 1% agarose gel.

RNA-Seq analyses were carried out using an Illumina NextSeq 500 platform (Illumina, San Diego, CA, United States).

qPCR was carried out as described by Botton et al. (2022) to validate RNA-Seq results. The gene *VvUBC28* (*VITVI19G00744*) adopted by Castellarin et al. (2007) was used for normalization. All the primers used in qPCR are listed in Supplementary Table 1.

### Bioinformatic and statistical analyses

Adaptor-trimmed RNA-Seq reads were mapped onto the PN40024.v4 version of the *Vitis vinifera* genome (Velt et al. 2023). A count matrix with merged read counts of each sample was obtained as described by Botton (2025) and used for the following analyses. Statistical analyses of differential gene expression were performed with the Bioconductor package *DESeq2* v1.38.3 (Love et al. 2014) with an FDR (False Discovery Rate) cutoff equal to 0.01 or 0.05, according to the contrasts, and a minimum fold change of 2. To remove genes with very low number of counts and high dispersions, the option “*Independent filtering of lower counts*” was selected. DESeq2 analysis and further functional analyses, such as the Pathview of hormone biosynthesis and signal transduction (Luo et al. 2017; Luo and Brouwer 2013), were carried out through the iDEP bioinformatic platform v2.01 (Ge et al. 2018) for a more integrated approach.

Upset plots were built using the R package *UpsetR* (Conway et al. 2017) to point out the number of genes in common between different groups of DEGs. This graphical representation is used in place of Venn diagrams when too many groups cannot be effectively represented with the canonical method (Lex et al. 2014).

Functional enrichments of multiple groups of genes are always difficult to represent graphically, due to the high number of GO (Gene Ontology) terms, their relative redundancy and the different depths of the terms (i.e., different levels of detail). Therefore, we adopted a simplification method based on “binary cuts” that was shown to perform better than other simplification tools. This method is implemented within an R package called *simplifyEnrichment* v1.14.1 (Gu and Hübschmann 2023) through the function *simplifyGOFromMultipleLists*. This function returns multiple graphics into a single image with bar charts on the left showing, for each cluster of enrichments, the number of significantly enriched GO terms for each contrast, a green–white–red heatmap in the centre to illustrate the significance of GO enrichments (an FDR cutoff equal to 0.05 was adopted), and a white–red heatmap showing the similarity matrix. For each cluster, the word clouds on the right display a semantic summary, with the size of each word corresponding to its representativity of the summarized enrichments.

Weighted gene correlation network analysis (WGCNA; Langfelder and Horvath 2008) was carried out with the R package *BioNERO* v1.6.1 (Almeida-Silva and Venancio 2022) to identify gene expression patterns associated with bud position and phenological stage. Genes with low levels of expression (min_exp = 10) and low variation were removed from this analysis, keeping only the 4,000 most variable. Outlying samples were removed with the “*pearson*” method and confounding artifacts were corrected with the “*PC_correction*” function (Parsana et al. 2019).

Hormone biosynthesis and signal transduction were studied by means of the Pathview tool and the KEGG (Kyoto Encyclopedia of Genes and Genomes) maps (Luo and Brouwer 2013; Luo et al. 2017) implemented in the iDEP workflow, and were approached by dividing data analysis according to the same criteria adopted in DEGs analysis, i.e*.*, by phenological phase, comparing the nodes, and by node, comparing the phenological phases.

Heatmaps were built with the R package *gplots v3.1.3.1*, using the *heatmap.2* function, the centroid method and Euclidean distance. Basic statistical analysis packages of R were used for Student’s *T* tests, ANOVA and various post hoc tests according to Vegro et al. (2016).

## Results

### Assessments of fertility and sample selection

**Fig. 1:**
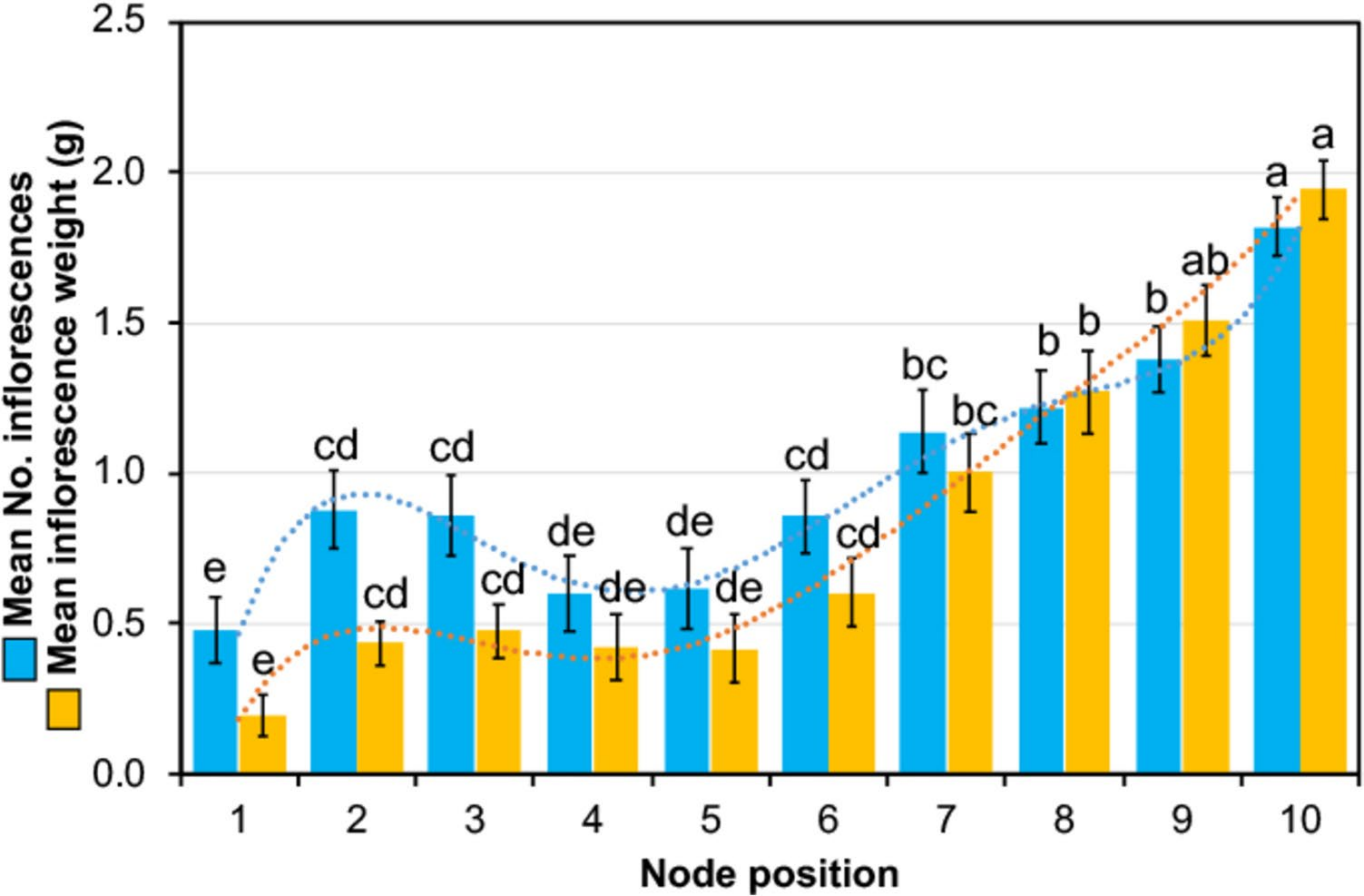
Fertility measurements on grapevine cv Merlot. Mean number of inflorescences (azure) and mean inflorescence weight (yellow) assessed for the first ten nodes along the cane of cv Merlot in 2024. Different letters indicate significant differences (see Materials and methods section for a detailed description of statistical methods), separately for each series of data (*P* < 0.05; *n* = 50 for inflorescence number; 24 < *n* < 91 for inflorescence weight). Bars indicate standard error. Dashed lines indicate the tendencies (polynomial, 5^th^ grade)

The fertility of the first ten nodes that were previously sampled for transcriptomic analyses was measured in 2024 to characterize the productivity patterns of Merlot grapevines and thus select the most representative nodes to be analysed through transcriptomic analysis (Fig. 1).

The mean number of inflorescences showed a generally increasing trend, supported by statistical analysis, from the first to the tenth node, with a slight decrease observed in the central at nodes 4 and 5. Inflorescence weight well-correlated with inflorescence number (Pearson’s index = 0.97) and displayed a more progressive increase from the basal to the distal buds, less marked in nodes 2 and 3. Both trends were fully overlapping with the 3-year fertility data measured on the same cv/rootstock in a different vineyard, particularly with 2024 data (Supplementary Fig. 2), thus confirming a reliable fertility pattern in this variety.

Based on these results, the three biological replicates of the buds from nodes 2, 5, and 10 were selected for transcriptome sequencing, for being representative of three different situations: medium fruitfulness of basal nodes, low fruitfulness of intermediate nodes, and high fruitfulness of apical nodes. The transcriptomic study was focused on phenological stages BBCH63 (anthesis; June 16th), BBCH77 (cluster closure; July 10th), and BBCH90 (harvest; October 10th), for covering critical stages of floral induction/transition/initiation (Carmona et al. 2002; Vasconcelos et al. 2009).

### Transcriptomic analysis

On average, the number of RNA-Seq reads was equal to 51 million per sample, with a mean overall alignment rate equal to 88.1% (Supplementary Table 2).

**Fig. 2:**
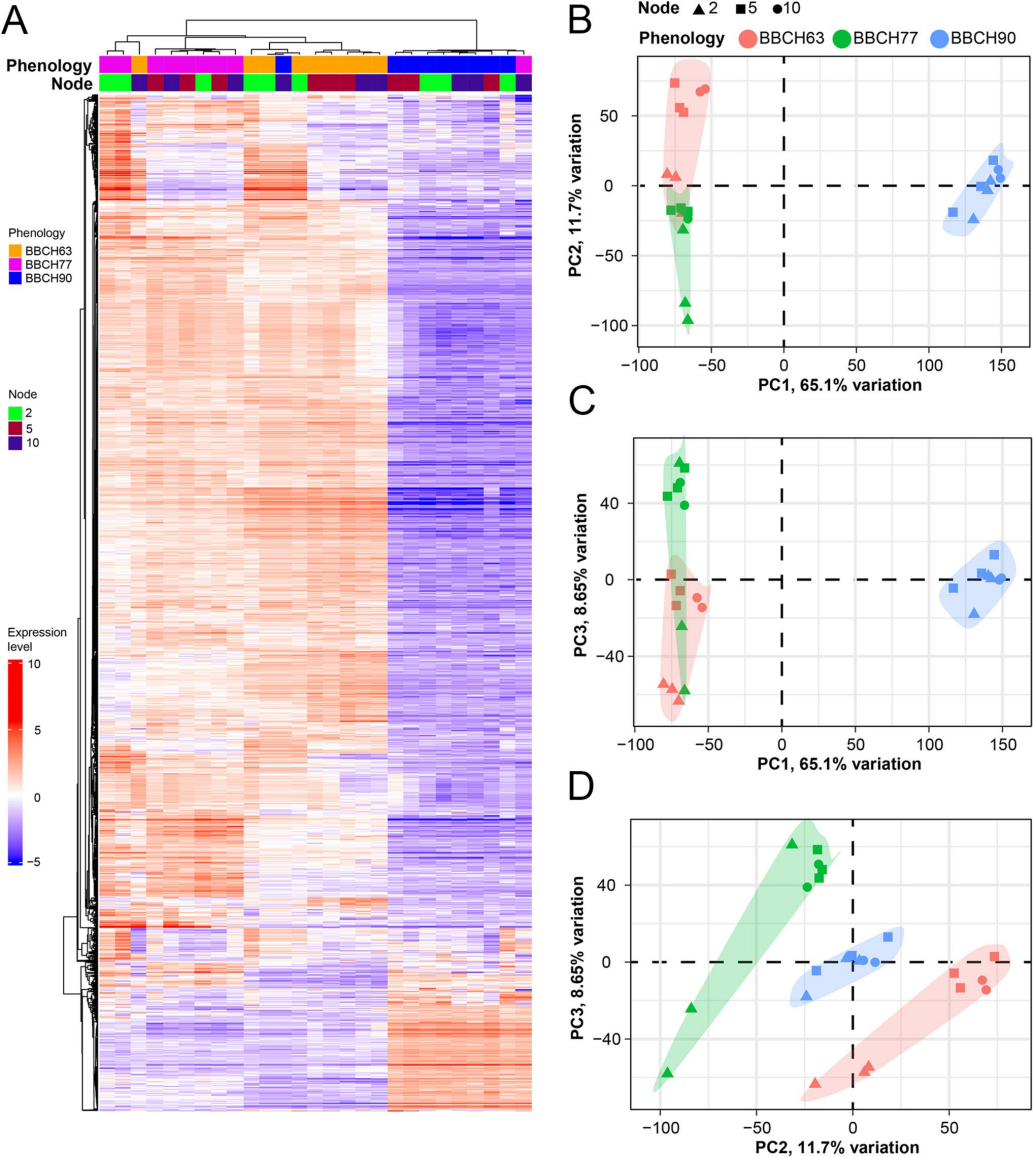
Hierarchical clustering and Principal Component Analysis (PCA) of samples. **A** Heatmap of the 3,000 most variable genes (including all biological replicates of each sample) hierarchically clustered according to Pearson distance, average distance and gene centering. The legends are reported on the left. **BD** PCA with principal components 1 and 2, 1 and 3, 2 and 3, respectively (excluding the outliers). The percentage of variance explained by each PC is shown in the axis label, while the legend is shown on the top

The heatmap and the hierarchical clustering built with the 3,000 most variable genes showed an excellent quality of replicates of the same samples, except for three replicates belonging to node 10 (Fig. 2A). These outliers were excluded from the statistical analyses for DEGs identification, to avoid biases. Principal Component Analysis (PCA) revealed that phenological stage is the main factor driving transcriptomic variation among samples. In Fig. 2B (PC1 vs PC2), PC1 (65.1% of the variance) clearly separates BBCH90 samples from BBCH63 and BBCH77, indicating major transcriptional changes associated with bud development. PC2 (11.7%) further contributes to the partial separation between BBCH63 and BBCH77. Within each phenological stage, samples show additional dispersion related to node position, although this effect is secondary to phenology. Figure 2C (PC1 vs PC3) confirms the dominant role of PC1, while PC3 (8.65%) enhances the separation between BBCH63 and BBCH77, particularly at early developmental stages. BBCH90 samples form a compact cluster, suggesting greater transcriptomic homogeneity at the end of the season. Finally, in Fig. 2D (PC2 vs PC3), the three phenological stages occupy distinct regions of the PCA space, allowing finer discrimination among stages. BBCH77 displays the highest dispersion, indicating increased transcriptomic heterogeneity during the floral transition phase, whereas BBCH63 and BBCH90 samples are more tightly clustered.

Overall, PCA highlights phenological stage as the primary determinant of gene expression variation, with node position contributing to intra-stage variability, especially during intermediate developmental phases.

### Identification of differentially expressed genes (DEGs)

**Fig. 3:**
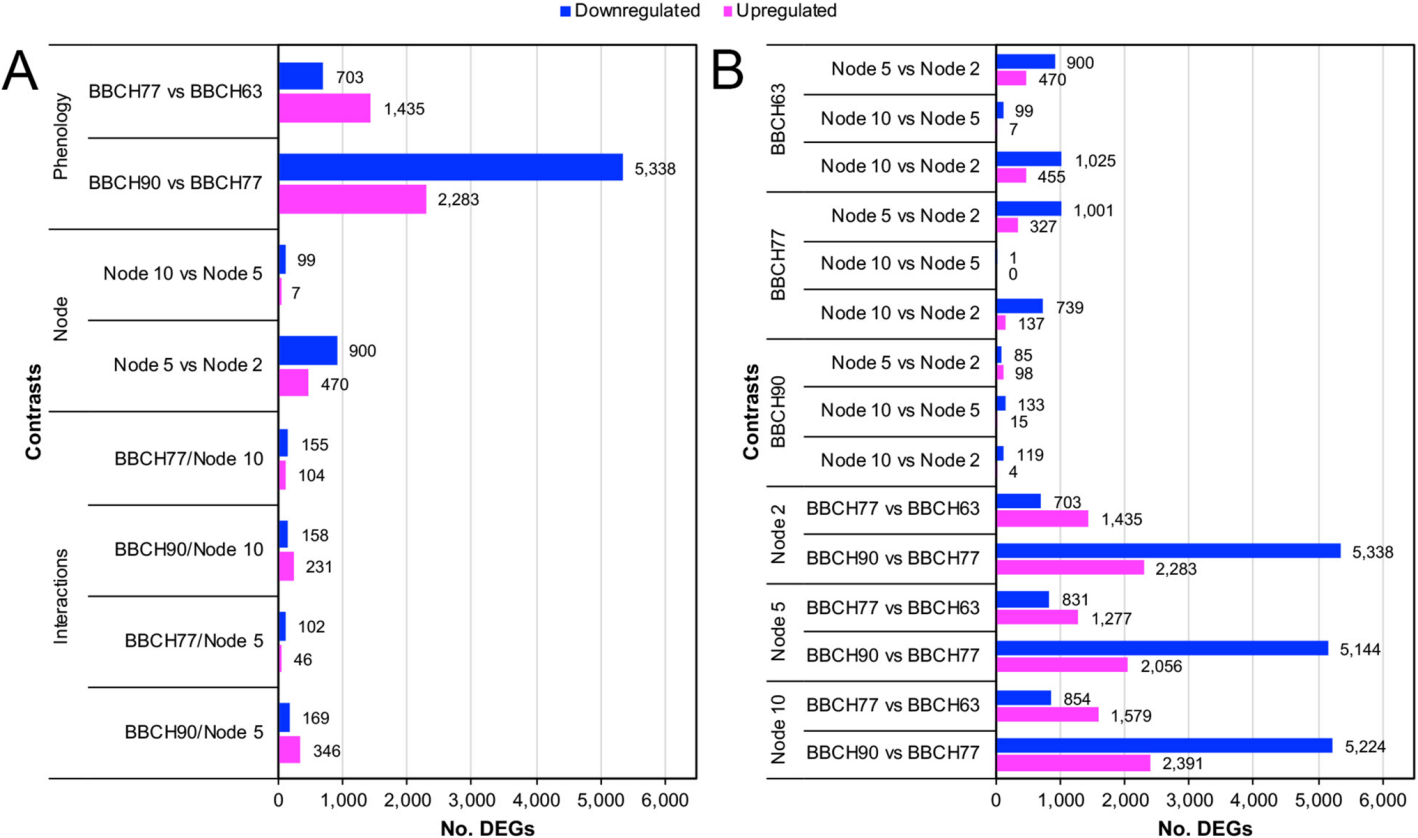
Number of differentially expressed genes (DEGs) in different types of contrast. **A** DEGs considering the single factors and their interactions. **B** DEGs considering the single contrasts between samples. FDR cutoff and minimum fold-change were equal to 0.05 and 2, respectively. The legend at the top of the chart shows the colours used for down- (blue) and up- (magenta) regulated genes

Significant transcriptional changes were observed between different phenological stages (Fig. 3A), especially between BBCH77 and BBCH90, with 5,338 downregulated and 2,283 upregulated genes. Between BBCH63 and BBCH77 the number of DEGs was lower, with 703 and 1,435 down- and up-regulated genes, respectively.

The positional comparisons showed lower numbers, with 900 and 470 down- and up-regulated genes in node 5 vs node 2, and only 99 downregulated and 7 upregulated genes in node 10 vs node 5. The number of DEGs resulting from the interaction between node and phenological phase was very low and will not be discussed.

Thereafter, the simple contrasts between samples were analysed, by grouping them either by phenological phase or by node, making the comparisons between nodes and between phases, respectively (Fig. 3B). The contrasts between the buds of nodes 5 and 2 were on average those with the highest number of DEGs, decreasing at BBCH90, and most of which included genes that were downregulated in node 5, while comparisons between nodes 10 and 5 were on average those with the lowest number of differentially expressed genes.

The contrasts between the same nodes at different phenological phases can provide further interpretations, showing closely similar regulatory patterns in buds of all nodes. Transcription in buds at 2nd node increased for 1,435 and 2,283 genes and decreased for 703 and 5,338, passing from BBCH63 to 77 and BBCH77 to 90, respectively. Buds at 5th node showed a similar trend, with 1,277 and 2,056 up-regulated, and 831 and 5,144 down-regulated genes, while those at 10th node pointed out 1,579 and 2,391 up-regulated, and 854 and 5,224 down-regulated genes.

According to the upset plots (Supplementary Fig. 3), 432 genes were downregulated in buds of node 5 vs node 2 at both BBCH63 and 77, while only 26 were downregulated in both BBCH77 and 90 in the same contrast. Comparing nodes 10 and 5, only 21 genes were in common between BBCH63 and 90. Among the upregulated, 106 were in common between BBCH63 and 77 for the contrast node 5 vs 2. A group of 6 genes was downregulated in buds of node 5 vs node 2 in all phenophases, such as *VITVI18G02191* encoding an ethylene-responsive transcription factor (ERF020), while only 1 gene was upregulated. Another example is given by the groups of 138 and 32 genes that were down- and up-regulated, respectively, in all the buds of the same node passing from a phenophase to the next. Among the upregulated, *VITVI11G00497*, encoding the auxin-responsive protein IAA9, was expressed at increasing levels in all buds of the same node during the entire experiment. An exhaustive list of these DEGs is provided in Supplementary Table 3.

Three genes were chosen for qPCR validation of RNA-Seq results carried out only at BBCH63. Two were selected among the DEGs, i.e., *VITVI11G00140*, encoding an ALP1-like protein with a possible indirect role in flowering (Liang et al. 2015), and *VITVI18G02191*, encoding an ethylene-responsive transcription factor (ERF020). The former was downregulated at node 5 vs node 2 only at BBCH63, while *ERF20* at all phenophases (Supplementary Table 3). The third gene was *VITVI04G00665*, encoding a CONSTANS-like protein (COL) shown to be expressed in latent buds (Almada et al. 2009), which was not differentially expressed according to RNA-Seq. This validation gave positive results, with largely overlapping expression patterns, indicating a strong reliability of RNA-Seq results (Supplementary Fig. 4).

### Functional analysis of DEGs (enrichment analysis)

**Fig. 4:**
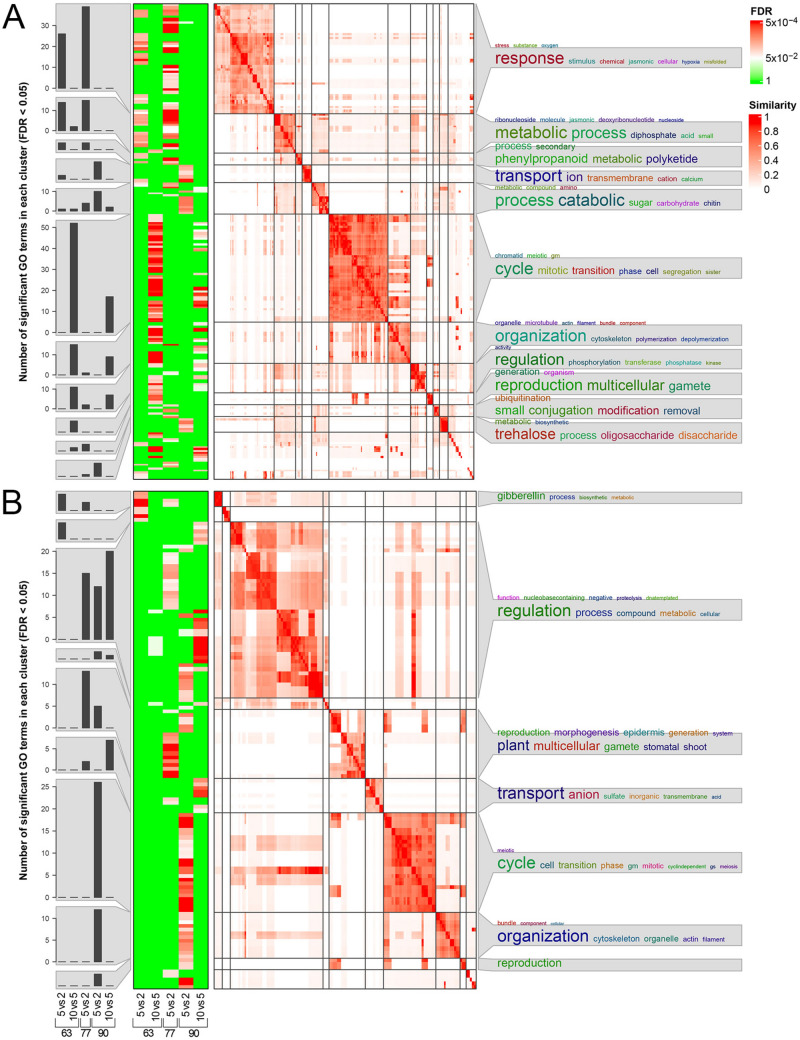
Enrichment analysis in the contrasts between nodes at different phenological phases on **A** downregulated and **B** upregulated genes. The bar charts in the first column show, when present, the number of enriched GO terms for each contrast at different phenophases (as indicated below the charts). The heatmaps in the second column display the statistical significance (FDR) of enrichments of the terms summarized through GO simplification on the right side of the figure. GO terms simplification was carried out with the R package *simplifyEnrichment* according to similarities among GO terms as summarized by the similarity matrices shown with the white-to-red colouration. Further details about the interpretation of these matrices can be found in the original publication (Gu and Huebschmann 2021). The colour scales for FDR and GO terms similarity are shown on the top right

**Fig. 5:**
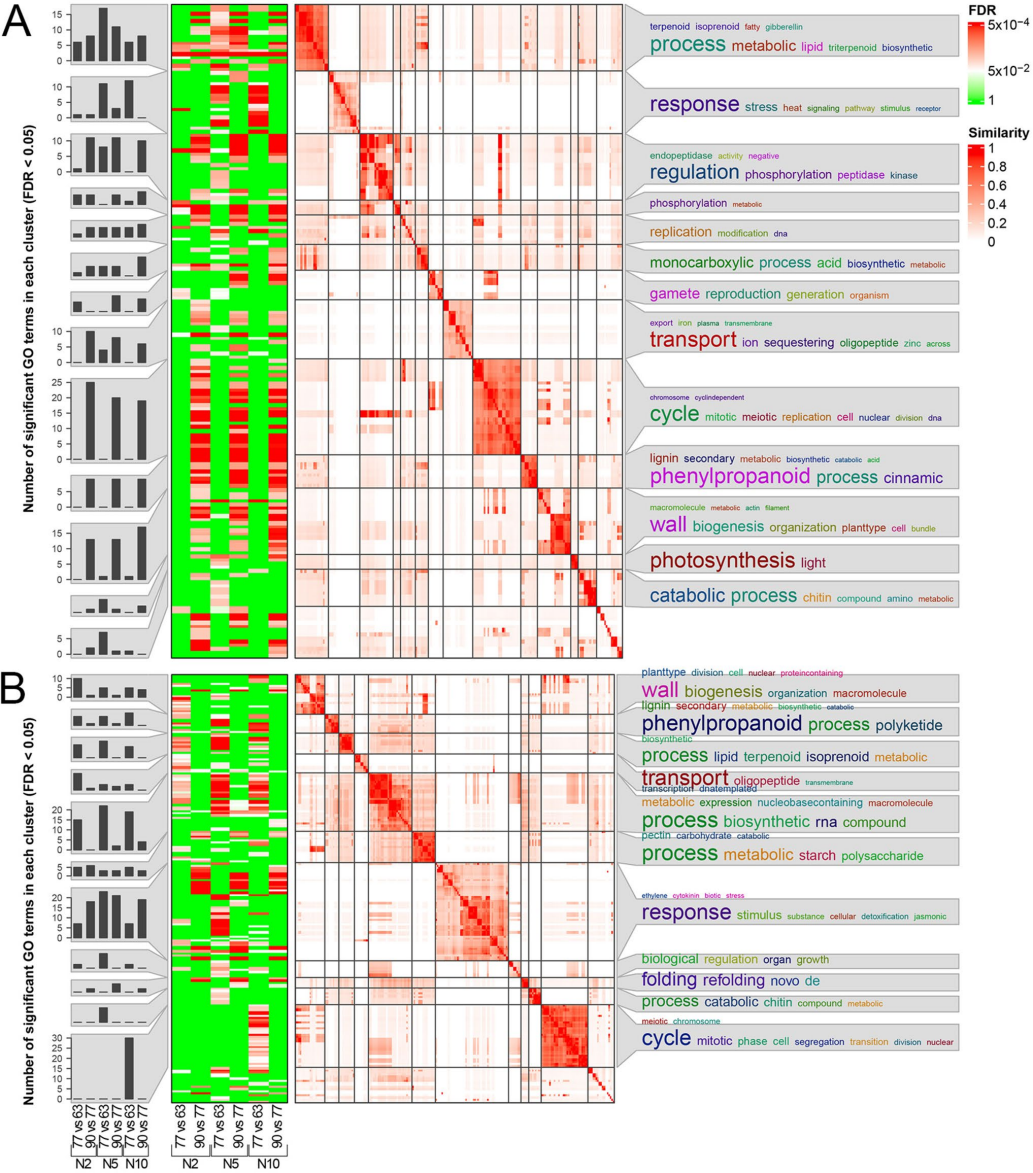
Enrichment analysis in the contrasts between phenological phases of different nodes on **A** downregulated and **B** upregulated genes. The bar charts in the first column show, when present, the number of enriched GO terms for each contrast at different nodes (as indicated below the charts). The heatmaps in the second column display the statistical significance (FDR) of enrichments of the terms summarized through GO simplification on the right side of the figure. GO terms simplification was carried out with the R package *simplifyEnrichment* according to similarities among GO terms as summarized by the similarity matrices shown with the white-to-red colouration. Further details about the interpretation of these matrices can be found in the original publication (Gu and Huebschmann 2021). The colour scales for FDR and GO terms similarity are shown on the top right

The *simplifyEnrichment* simplification tool allowed to obtain 103 and 130 enriched terms for the down- and up-regulated genes, which grouped into 11 and 7 clusters in the contrasts between nodes, respectively (Fig. 4), while 177 and 205 enrichments were clustered into 13 and 11 groups for the down- and up-regulated genes, respectively, in the contrasts between phenological phases (Fig. 5). On average, this tool allowed us to simplify the GO terms by a factor of 15, returning simple words derived from the GO biological process subvocabulary, herein used for being the most descriptive.

Concerning the downregulated genes (Fig. 4A), similar functions were found for the contrasts between nodes 5 and 2 at both BBCH63 and 77, with the three clusters represented by terms such as “*response*”, “*metabolic process*” and “*phenylpropanoid,*” including the highest number of DEGs. At BBCH90, the contrast between nodes 5 and 2 pointed out enriched functions dealing with ion transport, catabolic processes (mainly carbohydrates), and trehalose metabolism. Concerning the contrasts between nodes 10 and 5, enrichments were substantially shared at BBCH63 and BBCH90 (no enrichment at BBCH77), dealing with cell cycle, cytoskeleton organization and phosphorylation, indicating that these processes were more active in buds of 5th node than in position 10.

On the other hand, considering the enrichments of the upregulated genes (Fig. 4B), simplified into 7 clusters, a different pattern was observed with respect to the downregulated, with each contrast showing specific functions. The comparison at BBCH63 between buds at nodes 5 and 2 showed one enriched cluster, regarding only GAs biosynthesis and metabolism. The same contrast at BBCH77 displayed enrichments dealing with a general “*regulation*” and, interestingly, terms, such as “*shoot*”, “*reproduction*” and “*morphogenesis*”, in common with BBCH90. In the latter phase, additional enrichments were found regarding cell cycle, cytoskeleton organization, and “*reproduction*”, showing consistency with the downregulated genes. Finally, regarding the two contrasts between nodes 10 and 5 at BBCH63 and 90, the number of DEGs was very low in both situations and only the latter showed enrichments, such as “*regulation*”, once again, and anion transport.

The other series of enrichments displays the contrasts between phenophases within the same node. Starting from the downregulated genes (Fig. 5A) and considering the 2nd node, the contrast between BBCH77 vs 63 showed enrichments mainly dealing with biosynthesis and metabolism of lipids, terpenoids, isoprenoids, and GAs. In addition, the contrasts in node 5 showed enrichments dealing with response to stress (heat) and stimulus, regulation of phosphorylation, ion/oligopeptide transport, catabolic processes, and other minor terms, while in node 10 the enrichments dealt again with response to stress (heat) and stimulus, in common with node 5, and a few less numerous functions. The comparison BBCH90 vs 77 at node 2 was also enriched with terms related to biosynthesis and metabolism of lipids, terpenoids, isoprenoids, and GAs, which confirms previous observations. Moreover, this contrast was enriched with additional functions regarding regulation of phosphorylation, ion/oligopeptide transport, phenylpropanoid biosynthesis and metabolism, but above all cell cycle and cell wall biogenesis, the latter processes being in common with the same contrast of nodes 5 and 10.

The upregulated terms showed complementary and consistent results with respect to the previous ones (Fig. 5B). In the BBCH77 vs 63 comparison, enrichments showed a similar trend in all nodes, with functions, such as cell wall biogenesis, phenylpropanoid biosynthesis and metabolism, biosynthesis and metabolism of lipids, terpenoids and isoprenoids, oligopeptide transport, biosynthetic processes, starch metabolism, and response to stimulus (including jasmonate, cytokinins and ethylene). Most of these biological processes were downregulated in node 10 vs 5 in the same timeframe and are now upregulated in node 5 vs 2, meaning that node 5 is mainly characterized by these transcriptional patterns. Nevertheless, at node 10 in the same stage comparison, the terms regarding the cell cycle were enriched as well, consistently with what shown above. Finally, regarding the contrast BBCH90 vs 77, few enrichments were pointed out mainly for node 10 that deal with cell wall biogenesis and organization, biosynthetic processes and starch metabolism. The only cluster of enriched terms in common among the three nodes in this contrast dealt with response to hormonal stimulus (jasmonate, cytokinins and ethylene). However, while the number of significant GO terms dealing with these biological processes showed a peak in buds of node 5 in BBCH77 vs 63, with the other nodes showing a significantly lower number of enrichments (Fig. 5B), the same numbers in the contrast BBCH90 vs 77 were constantly high in all nodes (~ 20 terms).

### WGCNA analysis

**Fig. 6:**
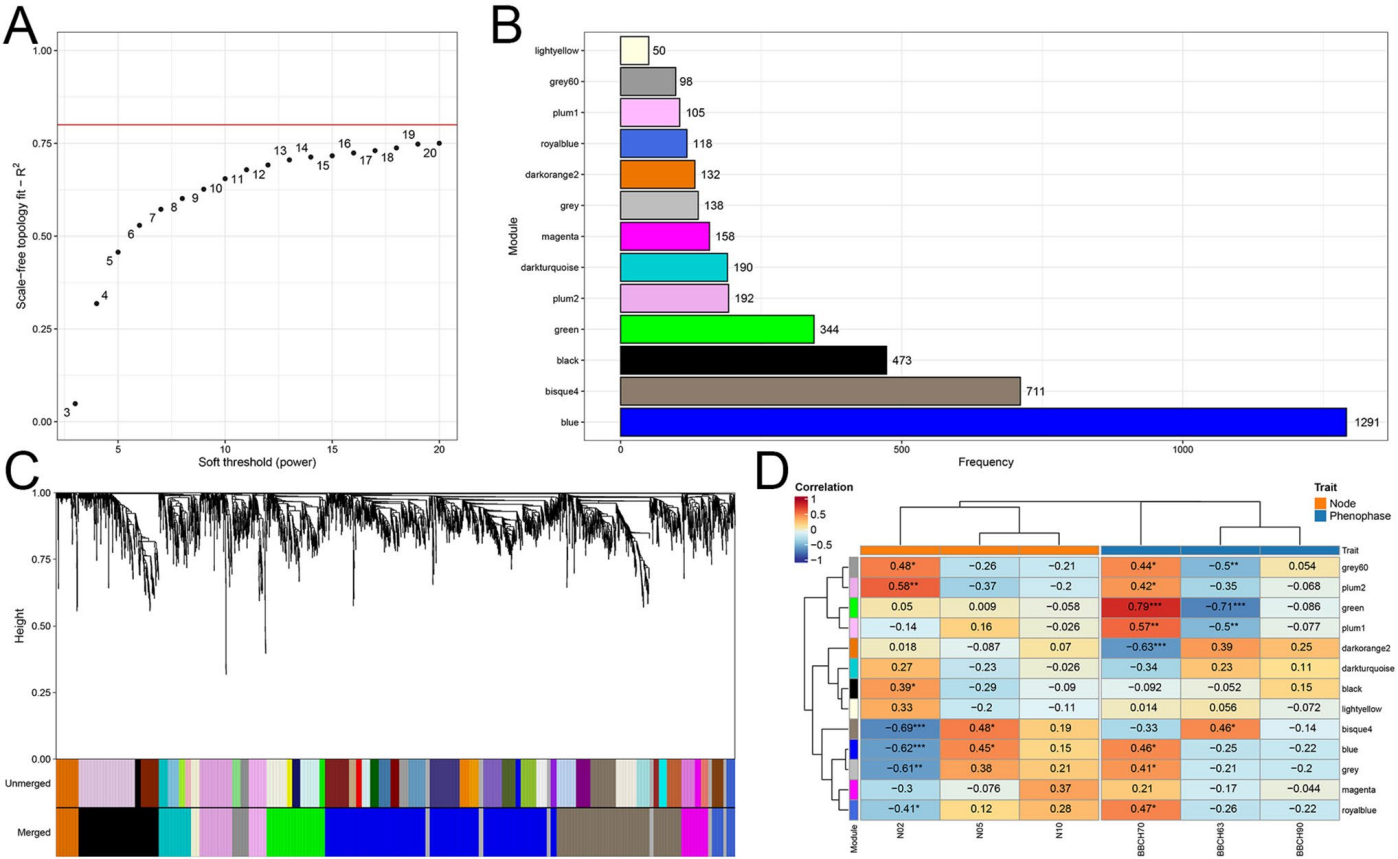
Weighted correlation network analysis (WGCNA) of the different bud samples. **A** Analysis of the scale-free fit index, with the red horizontal line marking the chosen cutoff (20). **B** Colours and number of genes for the WGCNA modules identified. **C** Hierarchical cluster tree of co-expression modules based on WGCNA. Unmerged and merged modules are shown below the tree. The different colours correspond to different modules. **B** Module-sample correlation matrix. The number in each cell indicates the correlation index (either positive or negative) between the module gene expression and the node or phenological phase, according to the colour scale shown in the top of the matrix. Asterisks indicate the level of statistical significance (* = significant at *alpha* = 0.05; ** = significant at *alpha* = 0.01; *** = significant at *alpha* = 0.001; no asterisk: not significant)

With a soft threshold power equal to 20, which is the maximum value (Fig. 6A), genes were clustered into 13 modules, the most numerous of which was the “*blue*” with 1291 genes, followed by the other modules up to “*lightyellow*”, with 50 genes (Fig. 6B).

Most of the 13 modules obtained, shown in Fig. 6C, had expression patterns positively or negatively correlated either with the phenological stages BBCH63 and 77, or with the bud positions 2 and 5, with very significant statistics. None of them was correlated with BBCH90 and bud position 10.

Seven gene modules were correlated with bud position 2, three positively (“*grey60*”, “*plum2*”, and “*black*”) and four negatively (both “*bisque4*” and “*blue*” with very high statistical significance; “*grey*”, and “*royalblue*”). Among the positively correlated, the “*black*” module is enriched of gene functions dealing with “*regulation of transcription, DNA-templated*”, while among the negatively correlated, there were enrichments related to “*mitotic cell cycle*”, “*cellular oxidant detoxification*”, and “*response to light stimulus*” for “*bisque4*” module, and “*translation*”, “*regulation of transcription by RNA polymerase II*”, “*protein folding*”, “*peptidyl-serine phosphorylation*”, and functions dealing with protein catabolism, just to cite the most representative of the “*blue*” module (Table 1). Patterns of expression of these genes are shown in Supplementary Fig. 5.

**Table 1:** Gene ontology biological process (GO–BP) terms enriched in the WGCNA modules

GO ID	GO term	No. Genes	No. Bkg genes	*p* value	FDR	Module
GO:0007015	actin filament organization	4	15	0.00019	0.009149	bisque4
GO:0098869	cellular oxidant detoxification	7	72	0.000636	0.026805	bisque4
GO:0015995	chlorophyll biosynthetic process	4	14	0.000142	0.007954	bisque4
GO:0000226	microtubule cytoskeleton organization	7	28	1.10E-06	7.44E-05	bisque4
GO:0000278	mitotic cell cycle	7	28	1.10E-06	7.44E-05	bisque4
GO:0015979	photosynthesis	5	38	0.000966	0.036189	bisque4
GO:0009768	photosynthesis, light harvesting in photosystem I	6	11	2.85E-08	9.60E-06	bisque4
GO:0018298	protein-chromophore linkage	8	34	3.08E-07	3.46E-05	bisque4
GO:0009416	response to light stimulus	8	32	1.85E-07	3.12E-05	bisque4
GO:0006355	regulation of transcription, DNA-templated	21	568	3.60E-05	0.012124	black
GO:0009073	aromatic amino acid family biosynthetic process	4	13	0.000994	0.025773	blue
GO:0002181	cytoplasmic translation	8	18	1.02E-07	1.73E-05	blue
GO:0006334	nucleosome assembly	5	14	0.000101	0.004247	blue
GO:0018105	peptidyl-serine phosphorylation	6	31	0.000814	0.023239	blue
GO:0010499	proteasomal ubiquitin-independent protein catabolic process	6	9	1.86E-07	2.09E-05	blue
GO:0043161	proteasome-mediated ubiquitin-dependent protein catabolic process	10	42	2.18E-06	0.000184	blue
GO:0006457	protein folding	9	72	0.001263	0.03041	blue
GO:0050790	regulation of catalytic activity	10	65	0.000123	0.004619	blue
GO:0006357	regulation of transcription by RNA polymerase II	13	127	0.000827	0.023239	blue
GO:0000027	ribosomal large subunit assembly	5	11	2.55E-05	0.00172	blue
GO:0042254	ribosome biogenesis	5	13	6.68E-05	0.003218	blue
GO:0006412	translation	31	150	4.34E-15	1.46E-12	blue
GO:0006414	translational elongation	7	26	3.17E-05	0.001779	blue
GO:0006413	translational initiation	7	36	0.000292	0.009827	blue
GO:0051259	protein complex oligomerization	5	19	7.73E-09	1.30E-06	darkorange2
GO:0006457	protein folding	6	72	3.20E-07	2.16E-05	darkorange2
GO:0009408	response to heat	7	31	2.19E-11	7.40E-09	darkorange2
GO:0042542	response to hydrogen peroxide	5	24	2.78E-08	3.13E-06	darkorange2
GO:0009651	response to salt stress	5	38	3.15E-07	2.16E-05	darkorange2
GO:0000165	MAPK cascade	2	8	5.53E-05	0.018627	lightyellow

The “*bisque4*” and “*blue*” modules were positively correlated with bud position 5. All the modules showing significant correlations with node position displayed an opposite behavior in the two nodes analyzed, i.e., those positively correlated with node 2 were negatively correlated with node 5, and vice versa. Genes encoding transcription elements are more expressed in node 2 than 5 (and 10), and buds at 2nd node are concurrently less active in terms of cell cycle, response to light, and translation (along with a series of post-translational modifications). These differences persisted during the whole experiment, indicating that the buds at these positions follow different developmental trajectories.

Six modules were positively correlated with BBCH70 (“*grey60*”, “*plum2*”, “*green*”, “*plum1*”, “*blue*”, and “*royalblue*”). Among these six modules, only the “*blue*” showed significant functional enrichment.

Only the “*darkorange2*” module was negatively and very significantly correlated with BBCH70, with enrichments related to “*protein folding*”, “*response to heat*”, “*response to hydrogen peroxide*”, and “*response to salt stress*”. In addition, in this case, an opposite behavior was observed for the same modules in the previous phenophase, i.e., BBCH63, with statistical significance resulting from “*grey60*”, “*green*”, and “*plum1*” for the negatively correlated, and “*bisque4*” for the positively correlated. The modules “*grey60*”, “*plum2*”, “*bisque4*”, “*blue*”, “*grey*”, and “*royalblue*” are in common between the nodes and the phenological stages, indicating possible interactions between these two factors. For example, the genes belonging to the “*blue*” module are, on average, expressed at highest levels at BBCH70 in buds at node 5, those of the “*bisque4*” module in buds at node 5 at BBCH63 (Supplementary Fig. 5), and the genes of modules “*grey60*” and “*plum2*” in buds of node 2 at BBCH70.

### Hormone biosynthesis and signal transduction

Pathview analysis was performed on the biosynthesis and signal transduction pathways of the five major plant hormones. Results were represented in two complementary ways, grouping samples either by phenophase or by node, allowing concurrent visualization of temporal and positional variation.

**Fig. 7:**
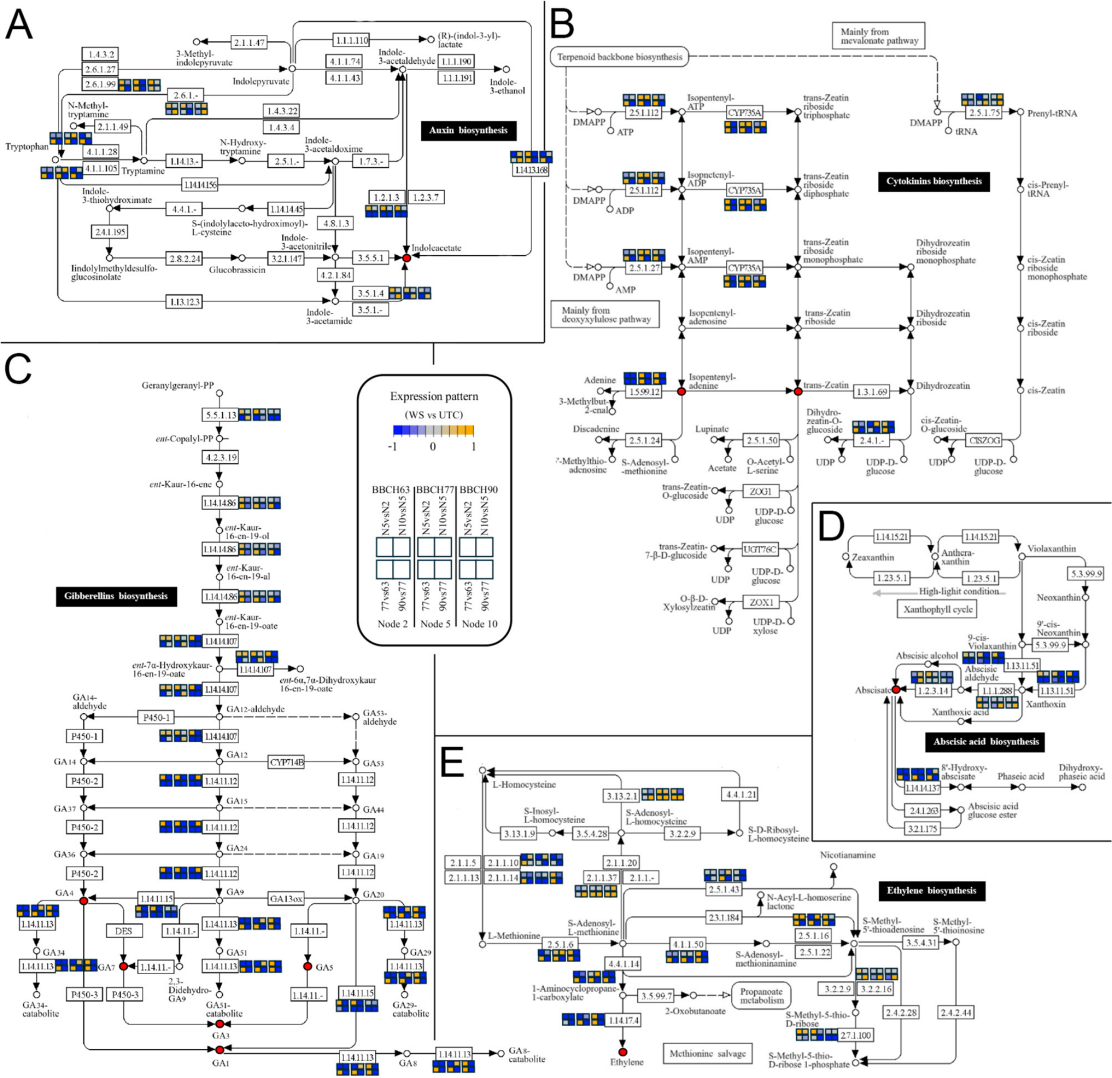
Pathview analysis of hormone biosynthesis through KEGG mapping. Maps were cropped leaving only the most relevant parts. **A** Auxin: map00380; **B** cytokinins: map00908; **C** gibberellins: map00904; **D** abscisic acid: map00906; **E** ethylene: map00270. The bioactive hormones are shown as red circles. The colour scale and a legend key are reported within the figure. Briefly, the expression pattern of each gene is shown through the twelve squares close to the gene name according to the colours and order shown in the legend

**Fig. 8:**
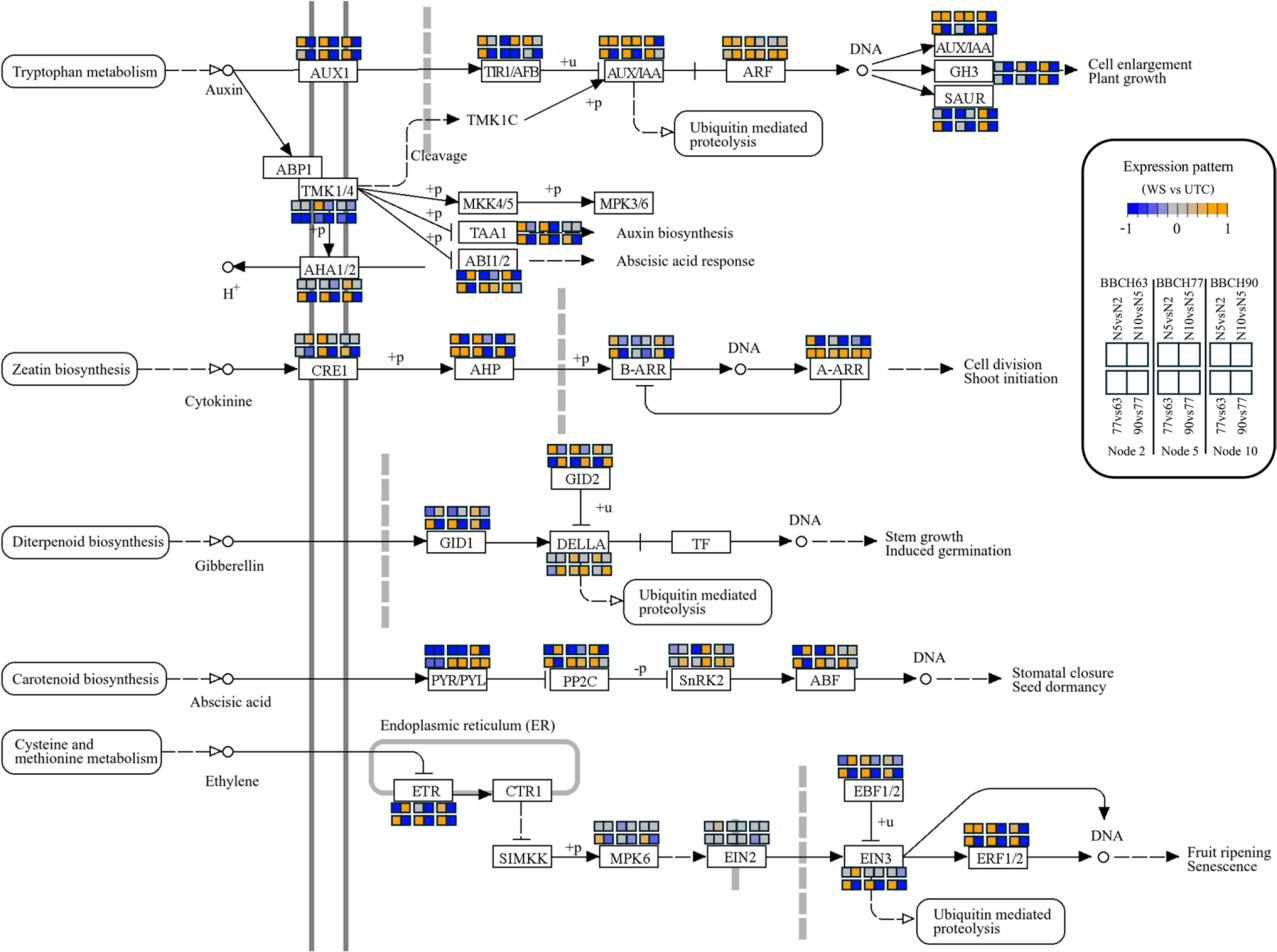
Pathview analysis of hormone signal transduction through KEGG mapping (map04075). The colour scale and a legend key are reported within the figure. Briefly, the expression pattern of each gene is shown through the twelve squares close to the gene name according to the colours and order shown in the legend

For auxin biosynthesis (Fig. 7A), attention was focused on the YUCCA (indole-3-pyruvate monooxygenase; EC 1.14.13.168) step, a major regulatory point in the pathway (Luo and Di 2023). During the first two phenophases, node 2 showed little variation, whereas YUCCA transcription increased at BBCH90. Nodes 5 and 10 displayed similar trends, with an initial increase followed by a decline. At BBCH63, node 5 showed the lowest expression levels, but this pattern reversed at BBCH77. At BBCH90, the highest YUCCA expression was observed in node 2 buds. Auxin signal transduction (Fig. 8) provided two main indications related to auxin homeostasis and signalling activity. Transcriptional profiles of biosynthesis genes (*TAA1* and *YUCCA*), transport components (*AUX1*), and conjugation enzymes (*GH3*) were largely consistent and indicated that buds at node 5 were more active than those at node 2, whereas buds at node 10 were less active than those at node 5. This differential activity was most pronounced at BBCH77 and decreased at BBCH90 in all nodes. *AUX*/*IAA* transcripts, commonly used as reliable indicators of auxin signalling (Abel et al. 1994), showed substantial overlap with these patterns when comparing phenophases. In positional contrasts, auxin signalling generally increased toward more apical nodes, except at BBCH90, when the shoot apical meristem was more distant from the sampled buds.

For cytokinins (Fig. 7B), IPT (isopentenyl transferase; EC 2.5.1.112), which catalyses the rate-limiting step (Kakimoto 2001; Takei et al. 2001), showed increased expression in all buds from BBCH63 to BBCH77, followed by a decline. *IPT* expression was similar across nodes at BBCH63, then followed a basal-to-apical increasing gradient at BBCH77 and finally decreased specifically at node 5 at BBCH90. *CYP735A* genes, encoding cytokinin hydroxylases responsible for trans-zeatin biosynthesis (Takei et al. 2004), displayed similar expression patterns. *Type-A ARR* (*RRA*) genes were more highly expressed in buds at node 5 than node 2 at BBCH63 and showed lower expression in node 10 at all phenophases (Fig. 8).

Gibberellins (GAs) biosynthesis (Fig. 7C) was evaluated through GA20ox (EC 1.14.11.12) and GA2ox (EC 1.14.11.13), which regulate biosynthesis and inactivation of bioactive GAs, respectively (Hedden 2020). *GA20ox* expression decreased from BBCH63 to BBCH90 in all buds. Positional comparisons showed higher expression in node 5 at BBCH63 and BBCH77, with a reversal at BBCH90. *GA2ox* transcripts increased from BBCH63 to BBCH77 in all nodes, then declined. Across nodes, an initially decreasing gradient from node 2 to node 10 was followed by slight increases at node 10 (BBCH77) and node 5 (BBCH90). For GA signalling (Fig. 8), *DELLA* genes were considered reliable indicators of GA response (Middleton et al. 2012). No differences were observed at BBCH63, whereas at BBCH77 and BBCH90 *DELLAs* were more highly expressed in node 5 than node 2, consistent with GA biosynthesis patterns. No significant differences were detected between nodes 10 and 5. Across phenophases, DELLA expression was highest at BBCH90.

For abscisic acid (ABA), the rate-limiting step catalysed by NCED enzymes (EC 1.13.11.51; Xiong and Zhu 2003) showed a progressive decline in transcript abundance across all buds, less pronounced at node 5, which maintained relatively higher expression levels (Fig. 7D). ABA signalling is known to contribute to bud dormancy induction at the end of the season (Zheng et al. 2015) and to growth inhibition during summer (Velappan et al. 2022). Because KEGG maps were not exhaustive for ABA signalling (Fig. 8), a targeted search identified three dormancy-associated genes in the grapevine genome. Their expression profiles suggested early activation of dormancy-related processes, particularly at nodes 5 and 10, already at BBCH63 (Supplementary Fig. 6).

Ethylene biosynthesis is regulated by ACC synthases (ACS; EC 4.4.1.14), encoded by a multigene family subject to transcriptional and post-translational control (Pattyn et al. 2021). *ACS* expression peaked at BBCH77 in all nodes (Fig. 7E). At BBCH63, a decreasing gradient from node 2 to node 10 was observed; at BBCH77, expression increased most strongly at node 10; at BBCH90, expression returned to patterns similar to BBCH63. Regarding ethylene signalling (Fig. 8), *ERF* genes showed overlapping patterns. At BBCH63, *ERF* expression increased from node 2 to node 10, whereas at BBCH77 and BBCH90 expression peaked at node 5 and declined at node 10. In all nodes, *ERF* transcripts increased from BBCH63 to BBCH77 and decreased at BBCH90.

### Expression analysis of flowering-related genes

**Fig. 9:**
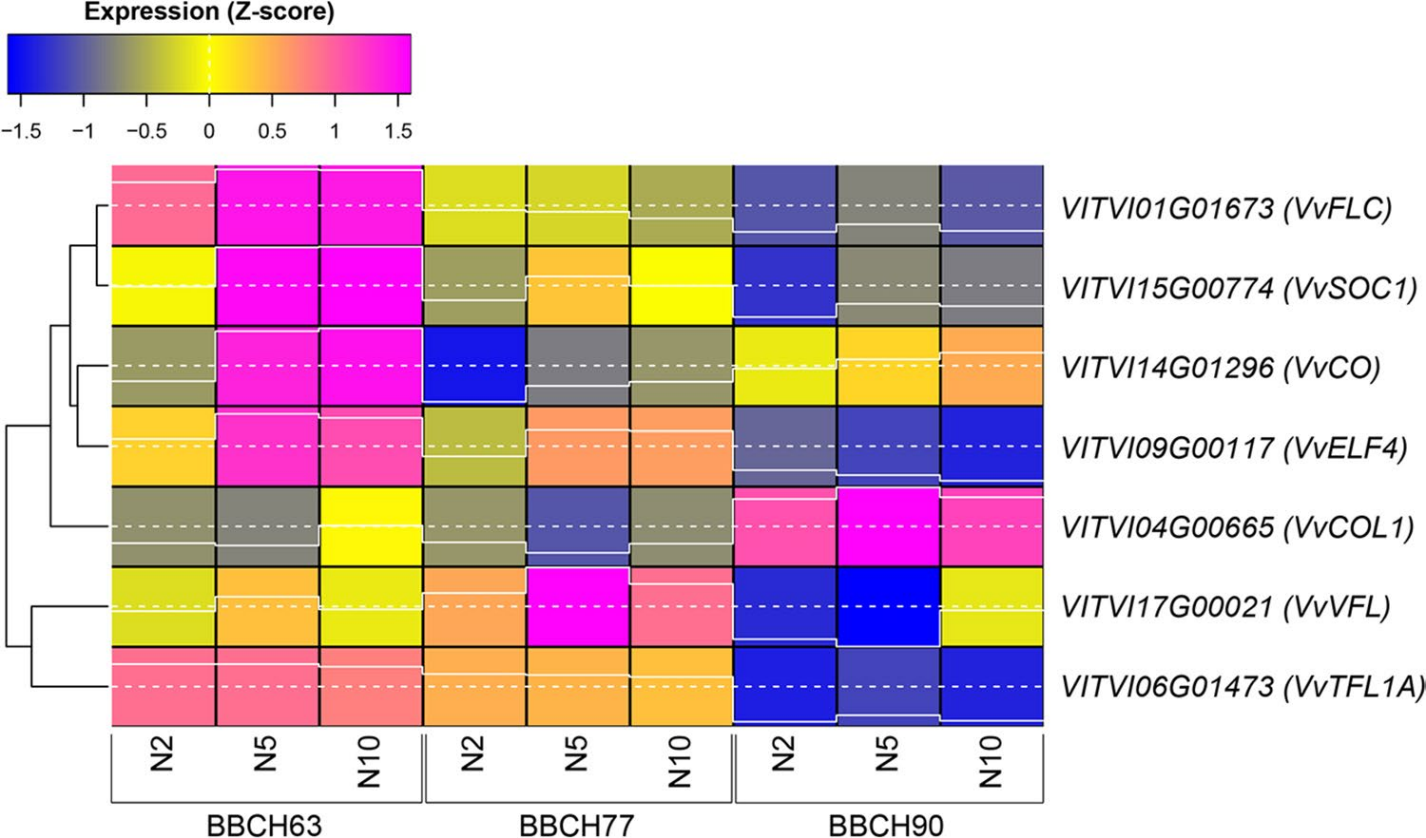
Expression heatmap of seven flowering-related genes across three phenological stages (BBCH63, BBCH77, BBCH90) and three bud positions (nodes 2, 5, and 10). Z-score normalized values were derived from data processed by iDEP after standardization. The gene names are shown in brackets after the gene ID. Expression levels range from blue (low) to magenta (high), as shown by the colour at the top left. Line traces within the boxes represent the expression levels. Hierarchical clustering groups genes according to similarities in their temporal expression patterns

Based upon previous studies (Almada et al. 2009; Carmona et al. 2007) and a search carried out on PN40024.v4 genome annotations (Velt et al. 2023), seven genes putatively involved in different steps of the flowering process, among which some of the know ‘floral integrators’, were selected to assess if their RNA-Seq expression patterns matched with previous publications and could effectively mark the ongoing flowering process in the present trial (Fig. 9).

Two main expression patterns plus an outlier emerged from this analysis. A first major cluster included *VvFLC* (*VITVI01G01673*), *VvSOC1* (also known as *VvMADS8*; *VITVI15G00774*), *VvCO* (*VITVI14G01296*), and *VvELF4* (*VITVI09G00117*). These genes generally displayed higher expression at BBCH63, particularly in nodes 5 and 10, followed by a decrease at BBCH77 and a further reduction at BBCH90. However, within this cluster, *VvCO* showed a distinct pattern: after declining at BBCH77, its expression slightly increased again at BBCH90 across nodes, unlike *VvFLC*, *VvSOC1*, and *VvELF4*, which continued to decline. This late-stage increase distinguished *VvCO* from the other genes in the same cluster and suggested a partially divergent temporal regulation, as previously pointed out by Almada et al. (2009).

A second cluster comprised *VvVFL* (*VITVI17G00021*) and *VvTFL1A* (*VITVI06G01473*), both characterized by relatively high expression at BBCH63 and/or BBCH77, followed by a pronounced downregulation at BBCH90 in all nodes. Their expression appeared strongly stage-dependent and less influenced by bud position, except a peak of expression at node 5 (BBCH77) for *VvVFL*. The general trend of their expression matched the decreasing patterns pointed out for *VvTFL1A* and *VvVFL* by Almada et al. (2009), Díaz-Riquelme et al. (2009) and Crane et al. (2012).

In contrast, *VvCOL1* (*VITVI04G00665*) behaved as an outlier. It showed comparatively low expression at BBCH63 and BBCH77 but exhibited a marked increase at BBCH90 across all nodes, indicating a distinct late-stage activation pattern. Again, this pattern of expression overlapped previous data published by Almada et al. (2009), who showed an increased expression of *VvCOL1* at dormancy inception.

## Discussion

### Bud fertility

Bud fertility patterns of cv. Merlot are largely consistent with published data, although most studies refer to other cultivars, such as Sauvignon Blanc (Eltom et al. 2014) or Sultana (Sommer et al. 2000), in which the increase in fruitfulness along the cane is less pronounced. Our results confirm that distal buds are more productive, likely due to greater exposure to light and higher temperatures, resulting in reduced competition for nutrients. This is consistent with previous studies showing that light availability is a key determinant of bud fertility and that distal buds, receiving more intense illumination, tend to be more fruitful (Huglin and Schneider 1998; Shaulis and May 1971; Smart 1985). In addition, reduced shoot crowding has been shown to improve light penetration and enhance bud fertility (Smart et al. 1982), while cane diameter and vigour have been identified as important contributing factors, with optimal light exposure being critical for inflorescence primordia induction and development (Vasconcelos and Castagnoli 2000). These findings may have practical implications for vineyard management. While traditional pruning to two buds is used to control vigour and maintain fruit quality, retaining additional buds could increase yield due to the higher fertility of distal positions, provided that overcropping risks are carefully managed. The observed variability in bud productivity further highlights the importance of canopy management, as practices that improve light exposure, such as shoot thinning and canopy positioning, can enhance fertility across bud positions. Maintaining appropriate vine vigour and cane diameter is, therefore, critical to maximize bud fertility.

### Positional and temporal transcriptomics

RNA-Seq data showed good transcriptome coverage across samples. PCA clearly separated samples by phenological stage, reflecting major developmental transcriptional changes (Díaz-Riquelme et al. 2012), and also by bud position, indicating a positional effect on gene expression. The distinct clustering of node 10 buds suggests position-specific transcriptional programs associated with higher fertility. DEG analysis identified BBCH77 as the most transcriptionally active stage and confirmed that phenological stage has a stronger impact on gene expression than bud position. Greater transcriptional divergence between nodes 5 and 2 than between nodes 10 and 5 indicates that node distance alone does not reflect physiological differences. Overall, transcriptomic differences among nodes progressively diminished toward the end of the season, consistent with convergence to a common physiological state and a gradual reduction of transcriptional activity during floral transition and initiation.

The functional enrichment analyses indicate that bud position and phenological stage jointly shape the regulatory programs underlying bud fertility. During the first two phenophases, buds at the 2nd node showed greater responsiveness to external stimuli and more active secondary metabolism than buds at node 5, suggesting higher developmental plasticity at basal positions. At BBCH90, enrichments related to carbohydrate and trehalose metabolism in node 2 buds are consistent with the key role of carbon availability in grapevine flowering (Lebon et al. 2008) and with trehalose-6-phosphate acting as a proxy for carbohydrate status (Wahl et al. 2013). In contrast, buds at node 5 were enriched for gibberellin biosynthesis and metabolism relative to node 2, in agreement with the inhibitory role of GAs in grapevine flowering (Boss and Thomas 2002) and with the reduced fertility observed at this position. Enrichment patterns further indicate that genes related to cell cycle and cytoskeleton organization were upregulated in node 5 compared with node 2 but downregulated relative to node 10, identifying node 5 buds at BBCH90 as the most transcriptionally active in terms of cell proliferation. However, this activity was accompanied by a late-stage slowdown of key physiological processes, including nutrient uptake. Stage-dependent enrichment analyses showed that GA biosynthesis is progressively deactivated in all buds and that from BBCH77 to BBCH90 there is a strong reduction in cell division and cellular structure biogenesis, indicating that bud architecture is largely established by the final phenophase. When positional and temporal patterns are integrated, buds at node 5 emerge as a distinct regulatory state characterized by competition among gibberellins, cytokinins, and ethylene. In these buds, the dominance of negative regulators, including ethylene, which acts as a floral repressor in Arabidopsis (Achard et al. 2007), likely over-rides cytokinin-mediated promotion of flowering, resulting in reduced fertility. In contrast, in buds at nodes 2 and 10, positive regulatory signals prevail, consistent with their higher fruitfulness.

WGCNA analysis further strengthened the idea that buds at different positions followed different developmental trajectories, but the most intriguing results were those coming from hormone biosynthesis and signal transduction analyses.

### Positional and temporal hormonal regulation

Results indicate that node 5 likely underwent a transient auxin biosynthetic activation at BBCH77, whereas node 2 showed increased auxin biosynthesis later, at BBCH90. Auxin biosynthesis in basal nodes appeared downregulated overall, likely reflecting their more advanced developmental stage. In contrast, in central nodes auxin biosynthesis seemed locally activated, possibly compensating for the increasing distance from the shoot apical meristem (SAM). In apical nodes, auxin may instead be largely supplied by the nearby SAM, reducing the need for local biosynthesis. A positive role of auxin during key developmental stages of grapevine buds, including floral initiation, has been proposed (Costantini et al. 2007) and is well-established in model species (Chandler 2011). In this context, the proximity of the SAM to apical nodes may partly explain the higher fertility observed at node 10, which could benefit from a sustained auxin flux from the apex.

Trans-zeatin biosynthesis was transcriptionally activated at BBCH77 in all nodes, following a basal-to-apical increasing gradient and declining at node 5 at the final timepoint. *RRA* genes are rapidly induced by cytokinins (D’Agostino et al. 2000) and are thought to mediate negative feedback regulation of CK signalling (Hwang et al. 2012), thereby reducing hormone sensitivity. The observed patterns are consistent with a more advanced developmental stage in basal buds and with a prominent, yet transient, role of cytokinins in node 5 buds at BBCH63. This cytokinin activity was not sustained at later stages, as previously suggested (Crane et al. 2012), potentially contributing to the lower fertility observed at this position. Overall, cytokinin signalling increased progressively across phenophases in all buds, consistent with the decreasing influence of SAM-derived auxin (Müller and Leyser 2011).

During the first two phenophases, node 5 displayed expression profiles compatible with higher gibberellin levels. Given the negative role of GAs in floral induction and transition (Boss et al. 2002), these data, together with GA signalling patterns, suggest activation of GA biosynthesis in node 5 at BBCH63 and BBCH77, followed by a later phase in which accumulated GAs were progressively deactivated by GA2-oxidases. Considering that buds at node 5 showed the lowest fertility, these findings are consistent with the inhibitory role of GAs in grapevine floral transition (Boss and Thomas 2002; Crane et al. 2012).

Analyses of ABA biosynthesis and response suggest that dormancy-related processes may have been activated earlier in node 5 buds compared with other positions (Velappan et al. 2022). Such early induction could have limited reproductive differentiation, contributing to reduced fertility.

Finally, ethylene biosynthesis appeared to increase at BBCH77, reaching its highest levels in node 10 buds. However, the distinct behaviour of node 5 in terms of ethylene signal transduction may have further influenced its fertility. Although the role of ethylene in perennials remains debated, it has been shown to act as a negative regulator of floral transition in Arabidopsis (Achard et al. 2007), and a similar regulatory effect cannot be excluded in grapevine.

### Expression of floral integrators and flowering-related genes

Information coming from the expression patterns of the known floral integrators and flowering-related genes allowed to consistently place the current study within the developmental series of events occurring during the flowering process in the first season. This analysis indicates that phenological stage exerted a stronger influence on flowering gene expression than bud position, while positional effects are more evident during early developmental stages and are gene-specific rather than uniform across the cluster. The consistency of expression patterns of all genes with the published data (Almada et al. 2009; Díaz-Riquelme et al. 2009; Crane et al. 2012) gave both general and specific indications, regarding the overall phenology and the relevancy of node position, respectively.

Concerning the phenology, BBCH63 could be identified as the floral induction phase, with all the important floral integrators, such as *VvFLC*, *VvSOC1*, and *VvCO*, expressed at maximum levels regardless of their specific positive or negative role in the flowering process. Among these genes, *VvFLC* (*Flowering Locus C*) has been well-studied in Arabidopsis, where it acts as a floral repressor in antagonism with FT and SOC1 in both vernalization and autonomous pathway (Michaels and Amasino 1999; Jiang et al. 2008; Deng et al. 2011). The observed expression patterns of *VvFLC* across development were consistent with previous results by Díaz-Riquelme et al. (2009) in buds of *Vitis vinifera* cv Tempranillo, showing an early upregulation of this gene from June to July, followed by a drop of its transcripts. Even though the role of this gene in grapevine, which does not need vernalization, remains to be elucidated, a possible accessory role within the autonomous pathway may be hypothesized as a sort of “plan B” to ensure flowering also under sub-optimal environmental conditions. Worthy to note that buds at node 2 showed a lower expression of all three genes of this cluster at BBCH63, indicating that the more advanced developmental stage of these buds likely over-rode the environmental control involved in floral induction. For a detailed study of the reciprocal dynamics of expression of these genes, a more intensive transcriptional time series would be necessary, but this was not the aim of the current study.

The following phenophase, BBCH77, could be placed on the following floral transition/initiation stage. This timepoint is characterized by the highest variability among nodes, consistently with the differences observed in the following year in terms of fertility. Worthy to note, *VvVFL* showed its highest expression at node 5, consistent with previous reports placing its maximum expression after *VvSOC1*, the latter being considered an important regulator of floral transition and development (Boss et al. 2002; Carmona et al. 2007; Almada et al. 2009; Pucker et al. 2020). *VvVFL* is the grapevine ortholog of Arabidopsis *FLO/LFY* and plays a central role in floral development (Carmona et al. 2002). It is expressed in shoot apical and lateral meristems that give rise to inflorescences, flowers, or tendrils, indicating functions in both flower initiation and maintenance of meristem indeterminacy (Poupin et al. 2011). Therefore, its high expression in node 5 buds may be consistent with a stronger and longer maintenance of meristem vegetative phase, which would prevent these buds from expressing their maximum reproductive potential under optimal environmental conditions.

Finally, the upregulation of both *VvCO* and *VvCOL1* at BBCH90 placed this phenophase immediately before buds’ dormancy inception. Indeed, besides the known link between *CONSTANS*-like genes and photoperiod (Putterill et al. 1995), which would justify their transient downregulation at BBCH77, an additional putative role in dormancy inception was hypothesized for both, especially for *VvCOL1* (Almada et al. 2009), thus reinforcing the placement of BBCH90 in the final phase of the annual cycle of the buds. In addition, *VvELF4*, a homolog of Arabidopsis *EARLY FLOWERING 4*, plays a crucial role in the circadian clock and photoperiodic flowering pathways in grapevine (Kamal et al. 2019). However, differently from *VvCO* and *VvCOL1*, its expression dropped at BBCH90, in parallel with the shortening of the days. To further strengthen this assumption, the expression of the four remaining genes was at very low levels at BBCH90, indicating the completion of floral induction, transition, and initiation phases, where these genes are mainly involved. Finally, a focus should be made also on *VvTFL1A*. It is a homolog of the *TERMINAL FLOWER 1* (*TFL1*) gene found in various plant species (Carmona et al. 2007), which acts as critical regulator of shoot architecture and flowering time, and typically functions as a flowering repressor, helping to maintain the plant's vegetative state and influencing how axillary meristems develop into either vegetative or reproductive structures (Moraes et al. 2019). This gene is crucial for controlling the balance between vegetative growth and reproduction, which ultimately affects grape yield and the structure of grape clusters. It is noteworthy that the expression pattern of *VvTFL1A* was completely dependent on the phenophase, regardless from bud position. In fact, this expression profile was not consistent with findings of Crane et al. (2012), who pointed out that in less fertile buds of cv Sugraone (a table grape cultivar) *TFL1A* is expressed at lower levels. However, since all the positive regulators of floral induction are expressed at high levels already at BBCH63, it is plausible that at this stage *VvTFL1A* expression was already decreasing, thus masking possible differences among buds at different nodes. To support this hypothesis, it is important to remember that its peak of expression in the bud may last for few days and is usually followed by a sudden drop (Crane et al. 2012).

Taken together these results indicate than the timing of the present study was chosen correctly, as most of the known markers of the flowering process in grapevine showed expression patterns at BBCH63 consistent with a bud developmental staging that varied from full floral induction of buds at nodes 5 and 10 to floral transition/initiation of buds at node 2.

## Conclusions

**Fig. 10:**
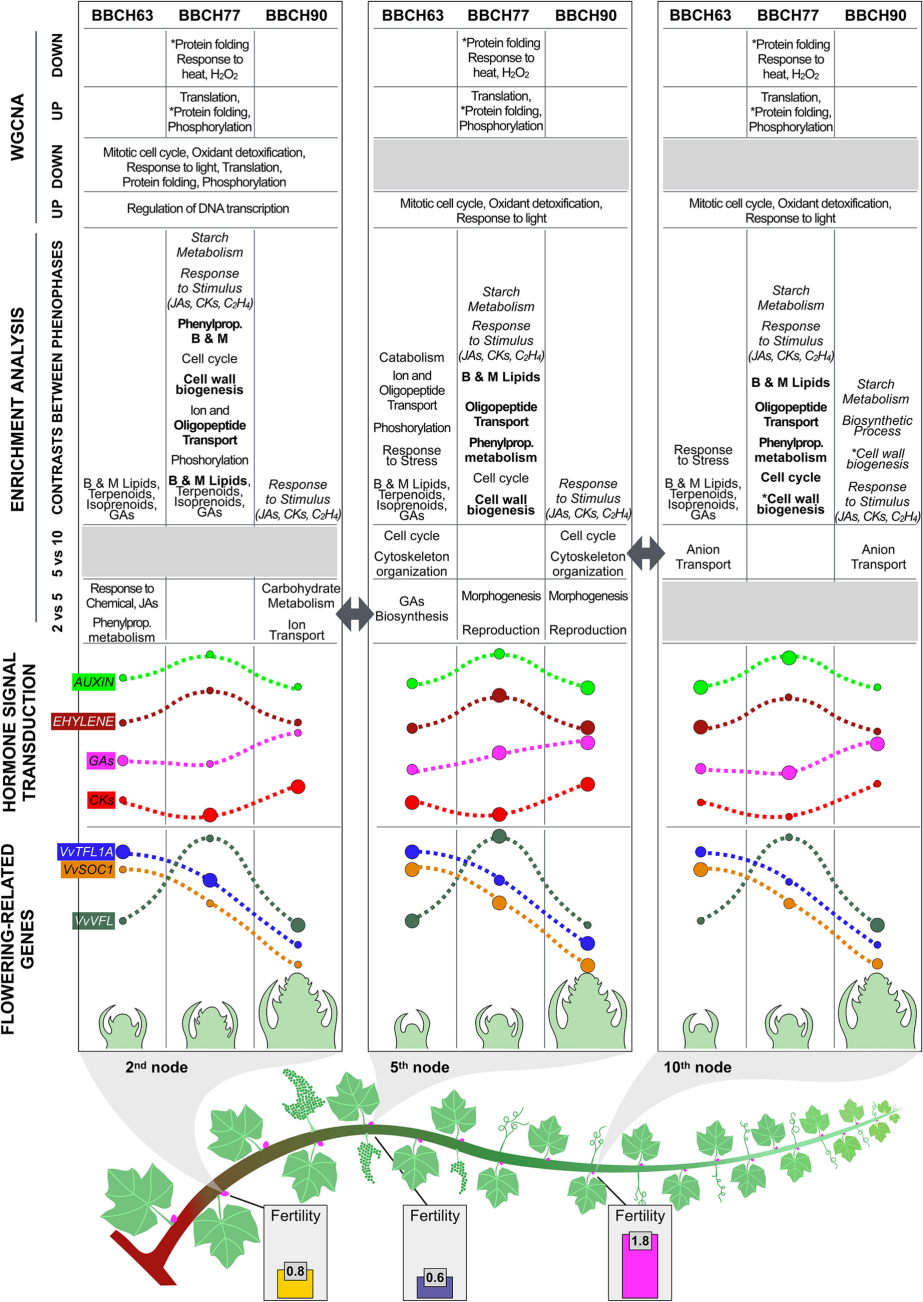
Model of grapevine bud development at three different nodes through different phenological phases (BBCH63, 77, and 90). Fertility is shown in the bottom. The charts of the flowering-related genes and hormone signal transduction show the trend through three stages (dashed lines). The size of the circles represents the trends in different nodes. The results of enrichment analyses in the comparisons between nodes are positioned in the phenological stage, where the biological processes are upregulated. In the comparisons between the phenological phases of the same node, the terms in regular font are upregulated with respect to the following phase, those in italics with respect to the previous one, while those in bold are upregulated with respect to both the other phase. Finally, regarding the WGCNA, the terms mark either the node or the phenological phase according to their position. Further explanations are given through the text

The comprehensive transcriptomic analysis of grapevine buds performed in this study provides new insights into the physiological and molecular mechanisms governing bud development and floral transition. Rather than focusing on individual genes, we applied complementary bioinformatic approaches to identify functional signatures associated with bud fertility. The convergence of results from differential expression, enrichment analyses, and network analysis allowed us to define the physiological background characterizing each node during development and to propose an integrative working model (Fig. 10) that may serve as a framework for future research on grapevine flowering.

Bud fertility followed an increasing gradient, with highest levels at node 10, intermediate levels at node 2, and lowest levels at node 5. This gradient represents a potential fertility, as measurements were taken after removal of lateral shoots, whose growth can inhibit flowering in subtended buds and partially mask intrinsic fruitfulness.

Flowering-related genes showed similar developmental trends across nodes, likely reflecting regulation by systemic inductive signals, such as FT, which integrates environmental cues, including light and temperature. However, relative expression levels differed among nodes. In particular, *VvVFL* reached its highest expression at node 5 at BBCH77. Given that *VvVFL* is normally expressed sequentially after *TFL1A* and *SOC1* (Almada et al. 2009; Crane et al. 2012) and is implicated in maintaining meristem indeterminacy (Carmona et al. 2002), its sustained or elevated expression may have interfered with timely floral initiation at node 5, consistent with observations in Arabidopsis overexpressing *VvVFL* (Carmona et al. 2007).

Hormonal signalling displayed similar developmental trajectories across nodes but differed in relative intensity. In buds at node 2, lower signalling activity of inhibitory hormones, such as gibberellins and ethylene, combined with enhanced cytokinin signalling during critical phases of floral induction and transition, may have compensated for partial developmental asynchrony with environmental conditions.

In contrast, buds at node 5 maintained high levels of inhibitory hormonal signalling through BBCH77 and BBCH90, even when cytokinin signalling increased. Enrichment analyses indicated upregulation of gibberellin biosynthesis at BBCH63 and of morphogenesis- and reproduction-related functions at later stages. Ion transport and high-energy metabolism were enhanced in nodes 2 and 10, whereas cell cycle and cytoskeleton organization—indicative of a less differentiated state—were more active at node 5 at BBCH63 and BBCH90 and were also specifically associated with node 5 in the WGCNA analysis. Together with the expression pattern of *VvVFL*, this regulatory configuration may have maintained primordia in a more undifferentiated state at node 5, reducing reproductive commitment despite responsiveness to environmental signals.

Buds at node 10, which displayed the highest fertility, showed hormonal profiles broadly comparable to node 2 but developed under more favourable environmental timing. Although mitotic cell cycle and light-response terms were associated with both nodes 5 and 10, the hormonal balance at node 10 appeared more permissive for floral transition. In these buds, exogenous factors (optimal light and temperature) and endogenous regulators (hormone balance and coordinated expression of flowering genes) likely acted synchronously to establish optimal conditions for floral induction, transition, and initiation.

Overall, bud fertility did not depend on a single determinant but emerged from a coordinated, stage-dependent balance between promotive and inhibitory hormonal cues, integrated with developmental progression and positional context. Although this study relied exclusively on transcriptomic data, future quantification of bioactive hormones and metabolomic profiling, as well as validation in cultivars with contrasting fertility gradients or under targeted agronomic treatments, will be essential to further refine and validate the proposed model.

## Supplementary Information

Below is the link to the electronic supplementary material.

Supplementary file1 (DOCX 1636 KB)

## Data Availability

The datasets supporting the conclusions of this article are available in the Gene Expression Omnibus (GEO) repository, under the accession number GSE277812 (https://www.ncbi.nlm.nih.gov/geo/query/acc.cgi?acc=GSE277812).
