# Supplementary material for: Transcriptional footprints associated with bud fertility in grapevine development (Vitis vinifera L.)
Source: Planta. 2026 Apr 8;263(5):128. doi: 10.1007/s00425-026-05000-3 (PMC13061797; doi:10.1007/s00425-026-05000-3)
Supplement: Supplementary file 1 — Supplementary file1 (DOCX 1636 KB) [file 425_2026_5000_MOESM1_ESM.docx]

## Supplementary material

**
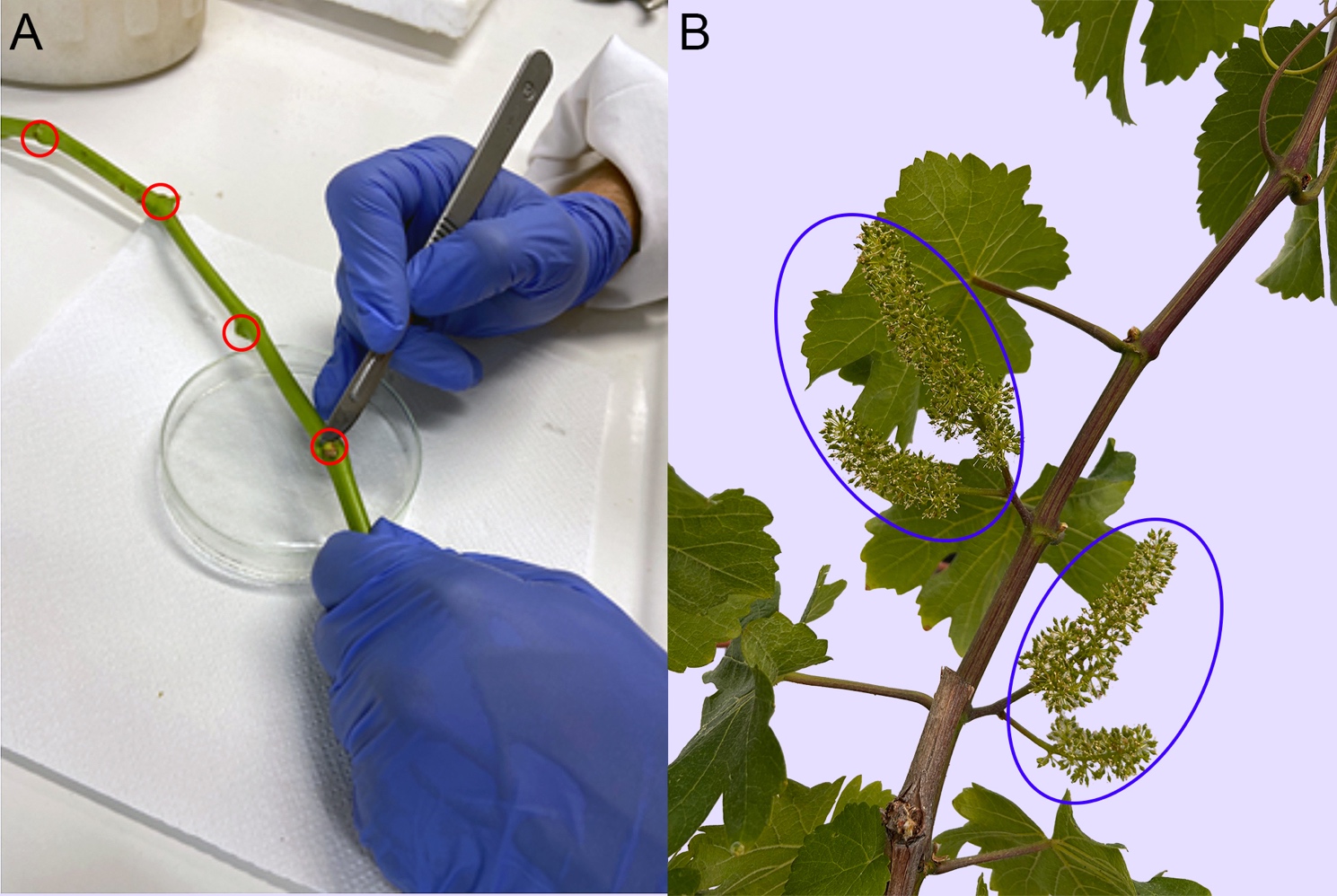
**

**Supplementary Figure 1.** Samples collected for molecular analyses and fertility measurements. A. For molecular analyses, buds were excised in 2023 with a scalpel from each node (red circles), immediately put in liquid nitrogen and stored at -80°C for following analyses. B. Fertility measurements were carried out in 2024 at BBCH57 phenological stage (‘*inflorescences fully developed; flowers separating’*; Lorenz et al. 1995) by counting and weighting the inflorescences (blue ellipses) collected in a single date (see Materials and Methods) from each shoot at each node of the cane; the picture is only representative of the samples collected.

**
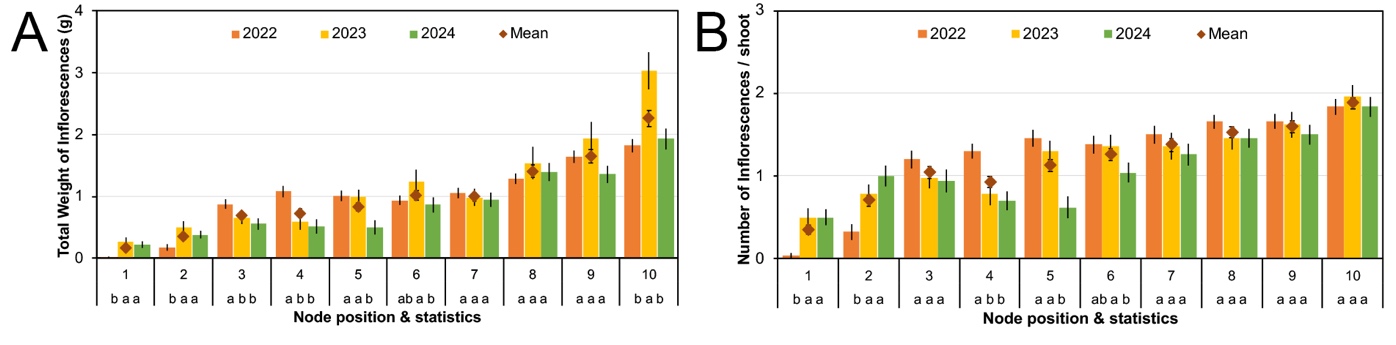
**

**Supplementary Figure 2.** Bud fruitfulness of cv Merlot in three consecutive years. Total weight (A) and number (B) of inflorescences per node measured in 2022 (orange), 2023 (yellow) and 2024 (green), and their mean value (brown diamond). Different letters below the nodes shows statistically significant differences between the years (*P* < 0.05). Clean and capped bars show the standard error (n = 50) in single years and mean values (n = 150), respectively.


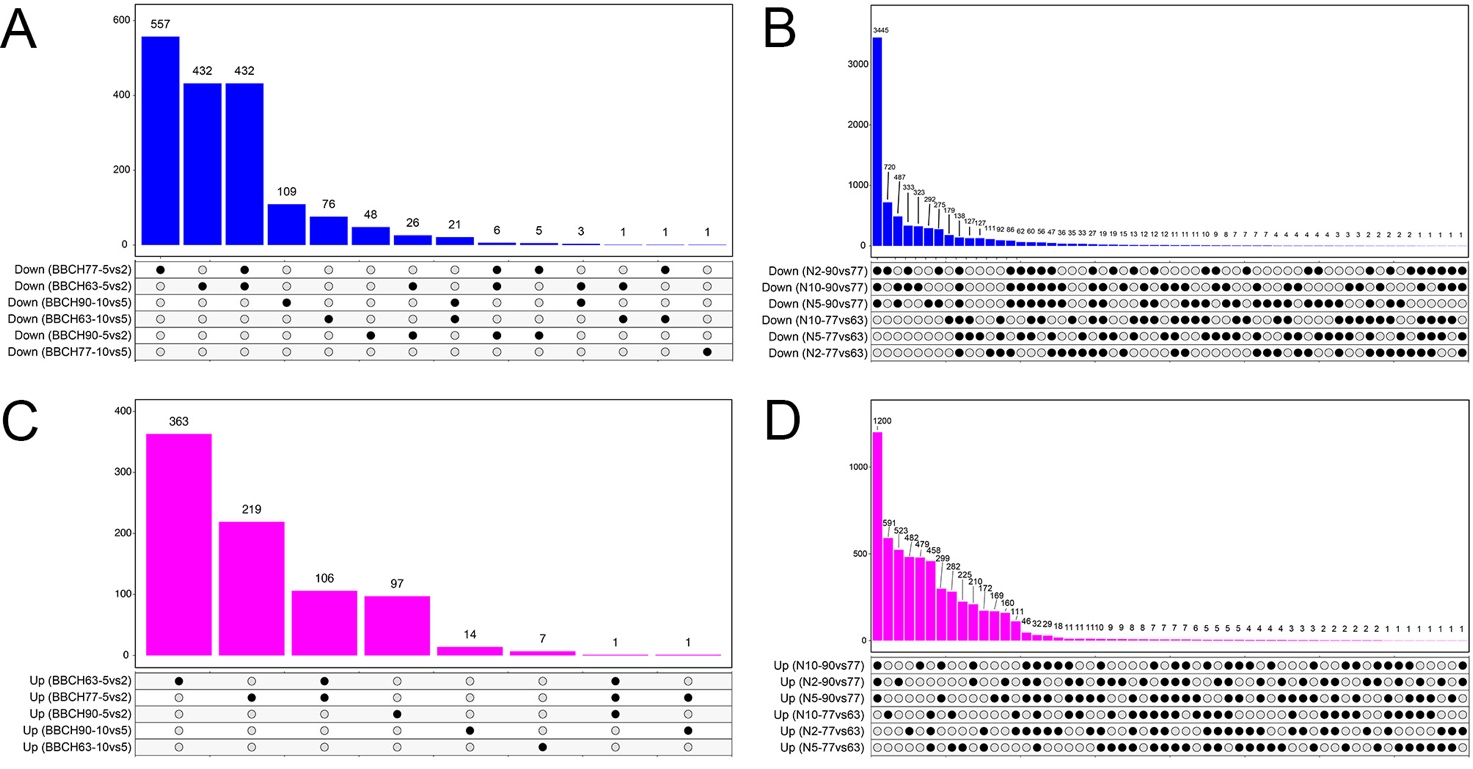


**Supplementary Figure 3.** Upset plots of DEGs. A, B) Down-regulated genes (blue). C, D) Up-regulated genes (magenta). A and C show the data related to the contrast between the nodes of the same phenological stage, while B and D show the contrasts between the same node in different phenological stages. The plot shows the intersections of the data in the combination matrix (bottom) and the columns show how many genes are in each intersection or only in one group. Plots were designed using the R package *UpsetR* (Conway et al., 2017).


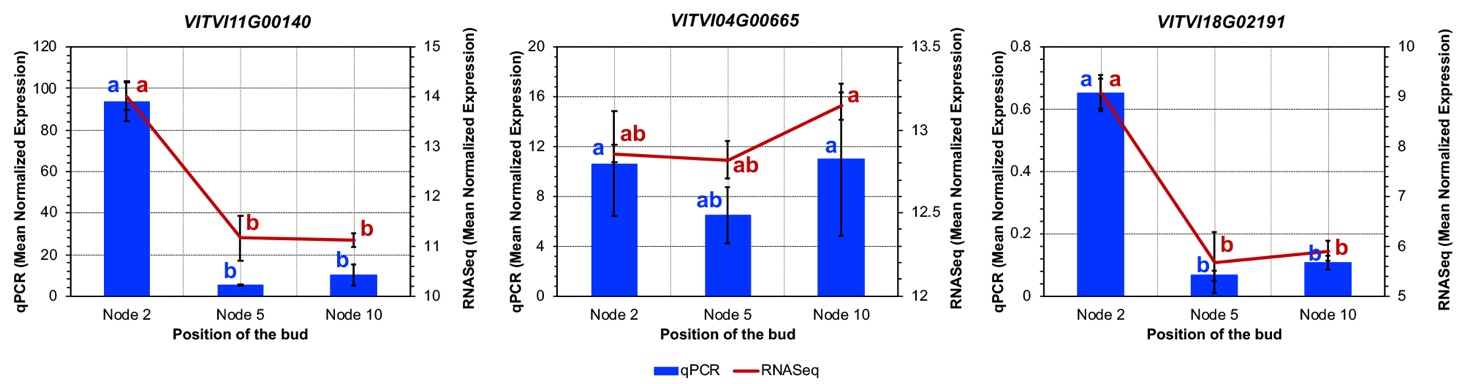


**Supplementary Figure 4.** Validation of RNA-Seq results through qPCR. Expression values of three genes were assessed at BBCH63 and are reported as arbitrary units of mean normalized expression for both the qPCR (blue lines) and RNA-Seq (red lines). The gene IDs are indicated at the top of each chart. Bars, where visible, indicate standard error. Ble and red letters indicate statistically significant differences (P $\leq$ 0.05) among the qPCR and RNA-Seq values, respectively.


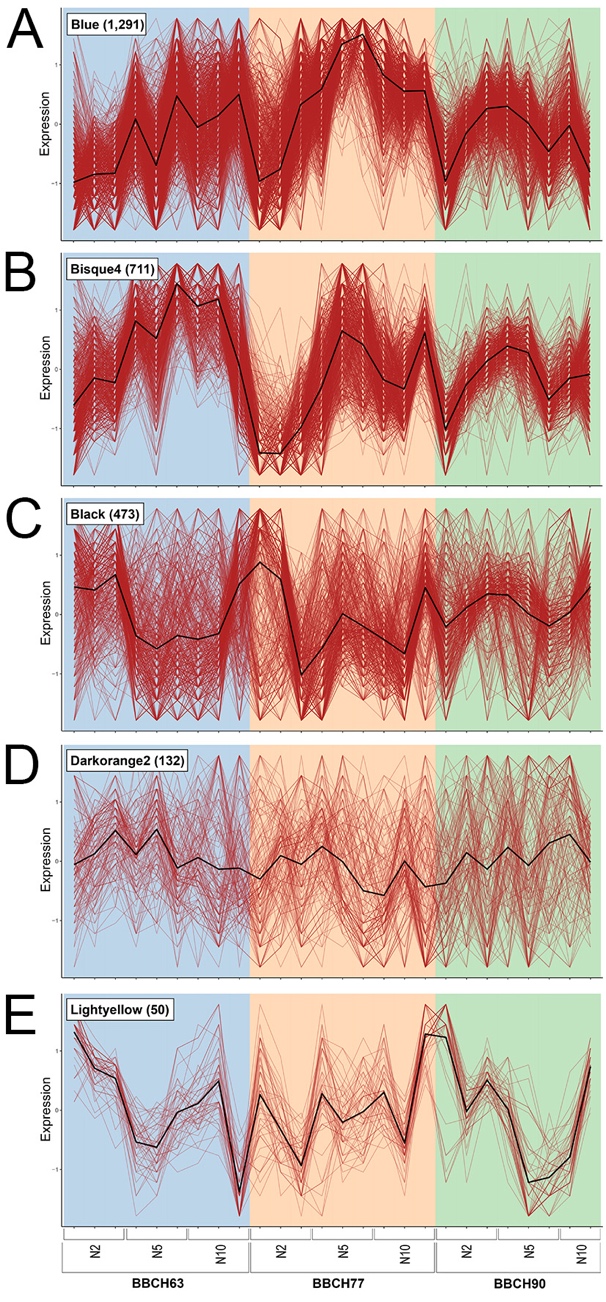


**Supplementary Figure 5.** Expression patterns of the genes belonging to relevant WGCNA modules. The numbers between brackets indicate the number of genes for each module. The position of samples (nodes and phenological phase) is shown below.


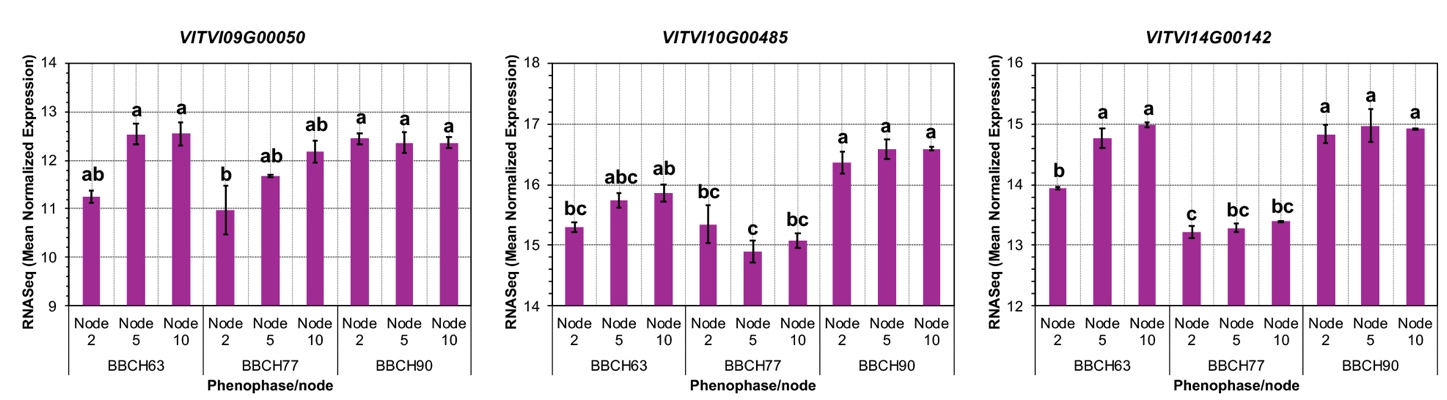


**Supplementary Figure 6.** RNA-Seq expression profiles of three dormancy-associated genes annotated as Dormancy-associated protein-like 4 (*VITVI09G00050*), Dormancy-associated protein-like 1 (*VITVI10G00485*) and Dormancy-associated protein-like 3 (*VITVI14G00142*). Bars indicate standard error (n = 3) and letters indicate statistically different values (*P*$\leq$0.05)

**Supplementary Table 1.** Sequences of the primers (Fw, forward; Rev, reverse) used for qPCR gene expression analyses.

| **Gene ID** | **Gene Name and abbreviation** | **Primer** | **Sequence (5'-3')** |
| --- | --- | --- | --- |
| *VITVI11G00140* | *Protein ALP1-like (ALP1)* | Fw | AAGTGGGCTTTGGATAATCAGAACG |
|  |  | Rev | CTCTCACTCTCCTCCATTTCACCA |
| *VITVI04G00665* | *Zinc finger protein CONSTANS-LIKE 4 (COL4)* | Fw | CTTTGACATGGATTTCTCAGGCTCC |
|  |  | Rev | CTGGCACGACTCCCACTTCTAA |
| *VITVI18G02191* | *Ethylene-responsive transcription factor (ERF020)* | Fw | TTTCCGTTCCCAGATAACACACTG |
|  |  | Rev | ATCACCTCCAGTAAAGCCTACCATC |
| *VITVI19G00744* | *Ubiquitin-conjugating enzyme (UBC28)* | Fw | CTATATGCTCGCTGCTGACG |
|  |  | Rev | AAGCCAGGCAGAGACAACTC |

**Supplementary Table 2.** Summary of RNA-Seq statistics in all replicates of all samples. The asterisks indicate the sample that were excluded from the differential gene expression analyses.

| **Phenology** | **Node position** | **Replicate** | **Total No. Reads** | **Reads not aligned concordantly** | **Reads aligned concordantly 1 time** | **Reads aligned concordantly > 1 time** | **Overall alignment rate** |
| --- | --- | --- | --- | --- | --- | --- | --- |
| BBCH63 | 2 | 1 | 48,620,094 | 14,469,897 | 33,142,031 | 1,008,166 | 89.8% |
| BBCH63 | 2 | 2 | 48,304,895 | 15,310,921 | 32,039,510 | 954,464 | 88.6% |
| BBCH63 | 2 | 3 | 48,573,244 | 48,573,244 | 32,785,296 | 991,839 | 89.3% |
| BBCH63 | 5 | 1 | 46,841,188 | 15,972,385 | 29,992,998 | 875,805 | 88.1% |
| BBCH63 | 5 | 2 | 49,311,999 | 14,859,324 | 33,569,770 | 882,905 | 89.7% |
| BBCH63 | 5 | 3 | 45,104,581 | 15,620,546 | 28,695,808 | 788,227 | 87.2% |
| BBCH63 | 10 | 1 | 54,410,035 | 17,366,083 | 36,072,952 | 971,000 | 89.0% |
| BBCH63 | 10 | 2 | 52,727,955 | 17,820,266 | 33,971,504 | 936,185 | 87.4% |
| BBCH63* | 10 | 3 | 52,157,763 | 11,853,984 | 39,299,093 | 1,004,686 | 90.9% |
| BBCH77 | 2 | 1 | 55,340,655 | 15,372,865 | 38,841,283 | 1,126,507 | 89.6% |
| BBCH77 | 2 | 2 | 53,576,680 | 14,469,412 | 37,775,099 | 1,332,169 | 89.3% |
| BBCH77 | 2 | 3 | 47,413,963 | 12,309,259 | 34,011,895 | 1,092,809 | 90.0% |
| BBCH77 | 5 | 1 | 46,496,635 | 11,019,151 | 34,583,036 | 894,448 | 90.9% |
| BBCH77 | 5 | 2 | 50,375,445 | 11,960,456 | 37,491,964 | 923,025 | 91.0% |
| BBCH77 | 5 | 3 | 62,868,940 | 15,959,144 | 45,735,822 | 1,173,974 | 90.7% |
| BBCH77 | 10 | 1 | 62,878,847 | 15,620,688 | 46,071,183 | 1,186,976 | 90.6% |
| BBCH77 | 10 | 2 | 54,464,102 | 13,213,203 | 40,236,437 | 1,014,462 | 91.3% |
| BBCH77* | 10 | 3 | 50,659,296 | 13,998,845 | 35,733,778 | 926,673 | 90.0% |
| BBCH90 | 2 | 1 | 55,908,600 | 16,355,756 | 38,533,399 | 1,019,445 | 86.3% |
| BBCH90 | 2 | 2 | 46,855,456 | 15,951,456 | 30,097,849 | 806,151 | 79.1% |
| BBCH90 | 2 | 3 | 49,434,743 | 16,258,578 | 32,332,948 | 843,217 | 81.6% |
| BBCH90 | 5 | 1 | 51,522,871 | 14,776,693 | 35,783,983 | 962,195 | 87.2% |
| BBCH90 | 5 | 2 | 52,142,791 | 14,359,200 | 36,727,108 | 1,056,483 | 87.2% |
| BBCH90 | 5 | 3 | 50,826,472 | 14,760,904 | 35,118,143 | 947,425 | 87.3% |
| BBCH90 | 10 | 1 | 48,658,350 | 15,465,833 | 32,355,875 | 836,642 | 86.0% |
| BBCH90 | 10 | 2 | 44,776,661 | 14,581,952 | 29,415,709 | 779,000 | 85.8% |
| BBCH90* | 10 | 3 | 48,954,737 | 16,419,089 | 31,677,109 | 858,539 | 86.2% |
|  |  | **Mean** | **51,081,741** | **16,099,968** | **35,262,651** | **970,127** | **88.1%** |

**Supplementary Table 3.** List of interesting marker genes with expression patterns in common among different contrasts. Group A: Downregulated in contrasts between node 5 and node 2, at all phenological phases; Group B: Upregulated in contrasts between node 5 and node 2, at all phenological phases; Group C: Downregulated in all the buds of the same node passing from a phenological phase to the next; Group D: Upregulated in all the buds of the same node passing from a phenological phase to the next.

| **Group** | **Gene ID** | **Uniprot Acc No.** | **Description** |
| --- | --- | --- | --- |
| Group A | VITVI05G02251 | RVW88564 | Endochitinase EP3 |
|  | VITVI06G00739 | XP_034687874 | cation/H(+) antiporter 15-like isoform X1 |
|  | VITVI10G00657 | XP_002264038 | MACPF domain-containing protein CAD1 isoform X1 |
|  | VITVI13G00131 | XP_010658205 | wound-induced protein 1 |
|  | VITVI16G01643 | RVW39394 | hypothetical protein CK203_117796 |
|  | VITVI18G02191 | XP_002276994 | ethylene-responsive transcription factor ERF020 |
| Group B | VITVI12G02721 | XP_010657977 | PREDICTED: uncharacterized protein LOC104881045 |
| Group C | VITVI00G04044 | XP_034688898 | RING-H2 finger protein ATL22-like |
|  | VITVI00G04529 | XP_002265991 | chalcone synthase |
|  | VITVI00G04601 | XP_002269451 | chalcone synthase |
|  | VITVI01G00234 | NP_001267887 | leucoanthocyanidin reductase 1 |
|  | VITVI01G00585 | XP_002281974 | very-long-chain 3-oxoacyl-CoA reductase 1 |
|  | VITVI01G01052 | XP_002268469 | myb-related protein 306 |
|  | VITVI01G01085 | XP_002266005 | PREDICTED: uncharacterized protein LOC100260888 |
|  | VITVI01G01657 | XP_002277578 | non-specific lipid transfer protein GPI-anchored 1 |
|  | VITVI01G01727 | XP_002263688 | somatic embryogenesis receptor kinase 1 |
|  | VITVI01G01851 | XP_019078452 | disease resistance protein RPM1 isoform X3 |
|  | VITVI01G04438 | C7AE94 | Anthocyanin-O-methyltransferase, VvAOMT |
|  | VITVI02G00238 | XP_002272570 | protein BRANCHLESS TRICHOME |
|  | VITVI02G00301 | XP_010660570 | putative transporter arsB |
|  | VITVI02G01331 | XP_003631374 | ABC transporter C family member 10 |
|  | VITVI02G01537 | XP_003631468 | geraniol 8-hydroxylase |
|  | VITVI02G04015 | RVX04067 | COP1-interactive protein 1 |
|  | VITVI03G00129 | XP_002277796 | actin-depolymerizing factor 5 |
|  | VITVI03G00391 | XP_002281159 | allene oxide synthase 3 |
|  | VITVI03G00393 | XP_002281201 | allene oxide synthase 3-like |
|  | VITVI03G00561 | XP_002285394 | probable cinnamyl alcohol dehydrogenase 1 |
|  | VITVI03G00568 | NP_001267849 | cinnamate beta-D-glucosyltransferase-like |
|  | VITVI03G00569 | XP_002274256 | limonoid UDP-glucosyltransferase |
|  | VITVI03G00598 | XP_002272036 | classical arabinogalactan protein 9 |
|  | VITVI03G01336 | XP_003631581 | auxin-induced protein 6B |
|  | VITVI03G01389 | XP_010647213 | proline-rich protein 4-like |
|  | VITVI03G01426 | XP_002281225 | probable flavin-containing monooxygenase 1 |
|  | VITVI04G00146 | XP_002279738 | serine/threonine-protein kinase STY46 |
|  | VITVI04G00508 | XP_002282808 | probable sucrose-phosphate synthase 1 |
|  | VITVI04G00509 | XP_010648678 | synaptotagmin-5 isoform X1 |
|  | VITVI04G00707 | RVW26893 | hypothetical protein CK203_104192 |
|  | VITVI04G00997 | XP_010648948 | GDSL esterase/lipase At5g33370 |
|  | VITVI04G01007 | XP_002268826 | GDSL esterase/lipase At5g33370 |
|  | VITVI04G01402 | XP_002273358 | aldehyde dehydrogenase family 3 member F1 |
|  | VITVI04G01475 | XP_002265520 | MLO-like protein 4 |
|  | VITVI04G01608 | XP_002276832 | probable plastid-lipid-associated protein 7, chloroplastic |
|  | VITVI04G01888 | CAN68521 | hypothetical protein VITISV_025154 |
|  | VITVI04G02232 | XP_010649410 | G-type lectin S-receptor-like serine/threonine-protein kinase LECRK3 |
|  | VITVI05G00961 | RVW88479 | Serine/threonine-protein kinase EDR1 |
|  | VITVI05G01266 | XP_002267838 | xyloglucan endotransglucosylase/hydrolase 2 |
|  | VITVI05G01288 | XP_002263422 | crocetin glucosyltransferase, chloroplastic |
|  | VITVI05G01605 | XP_002274083 | stearoyl-[acyl-carrier-protein] 9-desaturase, chloroplastic |
|  | VITVI05G01619 | XP_002274652 | stearoyl-[acyl-carrier-protein] 9-desaturase, chloroplastic |
|  | VITVI05G01620 | RVW24858 | Stearoyl-[acyl-carrier-protein] 9-desaturase, chloroplastic |
|  | VITVI05G02120 | XP_002267101 | CRIB domain-containing protein RIC4 |
|  | VITVI05G02201 | XP_019075669 | PREDICTED: uncharacterized protein LOC104879459 |
|  | VITVI05G04197 | XP_019075456 | glycine-rich protein 5 |
|  | VITVI05G04475 | RVW92004 | hypothetical protein CK203_030193 |
|  | VITVI05G04542 | RVW69311 | Stearoyl-[acyl-carrier-protein] 9-desaturase, chloroplastic |
|  | VITVI06G01290 | XP_002274065 | ABC transporter G family member 15 isoform X2 |
|  | VITVI06G01455 | XP_002263184 | alcohol-forming fatty acyl-CoA reductase |
|  | VITVI06G01513 | XP_002274217 | PREDICTED: uncharacterized protein LOC100258720 |
|  | VITVI07G00065 | XP_002274875 | ammonium transporter 3 member 1 |
|  | VITVI07G00279 | XP_010652025 | protein SRC2 homolog |
|  | VITVI07G00576 | XP_010652359 | 1-aminocyclopropane-1-carboxylate oxidase homolog 1 |
|  | VITVI07G01843 | XP_002275905 | alkane hydroxylase MAH1 |
|  | VITVI07G02185 | XP_002268411 | protein SRC2 homolog |
|  | VITVI07G02317 | XP_010652460 | glycine-rich protein 5-like |
|  | VITVI07G02318 | XP_010652461 | glycine-rich protein 5-like |
|  | VITVI07G02538 | XP_010646884 | probable glucan 1,3-beta-glucosidase A |
|  | VITVI07G02676 | XP_002281435 | L-ascorbate oxidase |
|  | VITVI07G02680 | XP_010652844 | PREDICTED: uncharacterized protein LOC104879924 |
|  | VITVI08G00100 | XP_010653170 | mini zinc finger protein 2-like |
|  | VITVI08G01250 | XP_010654244 | tropinone reductase homolog At2g29290 |
|  | VITVI08G01264 | XP_002271768 | diacylglycerol kinase theta-like |
|  | VITVI08G02368 | XP_003632717 | arabinogalactan peptide 13 |
|  | VITVI08G02377 | XP_002282798 | ras-related protein RABA4d |
|  | VITVI08G02398 | CAN69089 | hypothetical protein VITISV_009157 |
|  | VITVI08G04243 | XP_002271800 | PREDICTED: uncharacterized protein LOC100252758 |
|  | VITVI09G00183 | XP_010654524 | protein HOTHEAD |
|  | VITVI09G00253 | XP_010654591 | probable LRR receptor-like serine/threonine-protein kinase At1g05700 |
|  | VITVI09G00264 | XP_010654595 | GDSL esterase/lipase APG |
|  | VITVI09G00279 | XP_002283574 | hexokinase-2 |
|  | VITVI09G01345 | XP_003632925 | PREDICTED: uncharacterized protein LOC100852805 |
|  | VITVI09G01515 | XP_002274739 | auxin-binding protein ABP19a isoform X1 |
|  | VITVI09G01555 | XP_010654590 | senescence-induced receptor-like serine/threonine-protein kinase |
|  | VITVI09G02008 | XP_019077724 | beta-Amyrin Synthase 1 isoform X2 |
|  | VITVI10G00039 | XP_002264678 | beta-glucosidase 46 isoform X2 |
|  | VITVI10G00110 | RVW45901 | hypothetical protein CK203_068549 |
|  | VITVI10G01771 | XP_002279294 | protein E6 |
|  | VITVI10G04054 | RVW18815 | Ankyrin repeat-containing protein ITN1 |
|  | VITVI11G00247 | XP_010656249 | profilin-2 |
|  | VITVI11G01139 | XP_002269299 | putative clathrin assembly protein At5g57200 isoform X1 |
|  | VITVI11G01294 | NP_001267832 | Anthocyanidin 3-O-glucosyltransferase 2-like |
|  | VITVI11G01667 | XP_002272519 | PREDICTED: uncharacterized protein LOC100264996 |
|  | VITVI11G01671 | XP_002273403 | CASP-like protein 2C1 |
|  | VITVI12G00116 | XP_010657080 | probable LRR receptor-like serine/threonine-protein kinase At1g07650 |
|  | VITVI12G00213 | XP_034701387 | GDSL esterase/lipase At5g45910-like |
|  | VITVI12G00248 | XP_002277788 | fasciclin-like arabinogalactan protein 13 |
|  | VITVI12G00290 | XP_002275206 | early nodulin-like protein 1 |
|  | VITVI12G00292 | XP_002274522 | O-acyltransferase WSD1 |
|  | VITVI12G00480 | XP_002272560 | jasmonic acid-amido synthetase JAR1 |
|  | VITVI12G01605 | XP_010657601 | protein SODIUM POTASSIUM ROOT DEFECTIVE 1 |
|  | VITVI12G01624 | XP_002270950 | peroxidase 43 |
|  | VITVI12G04138 | XP_002276681 | GDSL esterase/lipase At2g04570 |
|  | VITVI13G00864 | XP_002265783 | cytosolic sulfotransferase 15 |
|  | VITVI13G01851 | XP_010659608 | protein UPSTREAM OF FLC isoform X2 |
|  | VITVI14G01317 | XP_002278423 | GDSL esterase/lipase 7 |
|  | VITVI14G01353 | XP_002277997 | PI-PLC X domain-containing protein At5g67130 |
|  | VITVI14G01881 | XP_002276619 | PREDICTED: uncharacterized protein LOC100267969 |
|  | VITVI14G01885 | RVX08702 | hypothetical protein CK203_010812 |
|  | VITVI14G01923 | XP_010661047 | PREDICTED: uncharacterized protein At5g39865 |
|  | VITVI14G02869 | XP_002284213 | non-specific lipid-transfer protein 2 |
|  | VITVI14G03025 | XP_019080237 | UDP-glycosyltransferase 90A1-like |
|  | VITVI14G04632 | XP_019080238 | UDP-glycosyltransferase 90A1-like |
|  | VITVI15G00396 | XP_002262833 | trans-resveratrol di-O-methyltransferase-like |
|  | VITVI15G01121 | XP_002274806 | rop guanine nucleotide exchange factor 3 isoform X1 |
|  | VITVI16G00275 | AGJ94050 | metacaspase-3 |
|  | VITVI16G00313 | XP_002278426 | cyclin-U4-1 |
|  | VITVI16G00713 | XP_002265534 | beta-fructofuranosidase, soluble isoenzyme I isoform X1 |
|  | VITVI16G00752 | XP_003634057 | stilbene synthase 3 |
|  | VITVI16G00793 | XP_002264723 | protein EXORDIUM-like 3 |
|  | VITVI16G00804 | XP_002267424 | 3-ketoacyl-CoA synthase 5 |
|  | VITVI16G01325 | PSS32938 | Transcription repressor like |
|  | VITVI16G01854 | XP_002267848 | protein HOTHEAD |
|  | VITVI16G02087 | RVW88980 | hypothetical protein CK203_029408 |
|  | VITVI16G04303 | XP_002265991 | chalcone synthase |
|  | VITVI17G00731 | XP_010663222 | formin-like protein 8 |
|  | VITVI17G01481 | XP_010663337 | PREDICTED: uncharacterized protein LOC104877445 |
|  | VITVI17G04271 | RVW20433 | Aldehyde oxidase GLOX |
|  | VITVI18G00018 | XP_010664042 | PREDICTED: uncharacterized protein LOC100264995 isoform X1 |
|  | VITVI18G00156 | XP_003634343 | ornithine decarboxylase |
|  | VITVI18G00683 | XP_002283279 | subtilisin-like protease SBT1.7 |
|  | VITVI18G01120 | XP_002275306 | tubulin beta-1 chain |
|  | VITVI18G01992 | RVW37858 | Protein NRT1/ PTR family 5.10 |
|  | VITVI18G02103 | XP_002279472 | beta-amyrin 28-oxidase |
|  | VITVI18G02126 | XP_034677108 | beta-amyrin 28-monooxygenase-like |
|  | VITVI18G02146 | XP_002278219 | GDSL esterase/lipase At1g71691 |
|  | VITVI18G02427 | XP_010665478 | potassium channel KOR2 isoform X1 |
|  | VITVI18G04021 | XP_010664895 | PREDICTED: uncharacterized protein LOC100255757 |
|  | VITVI19G00174 | XP_003634640 | UDP-glycosyltransferase 79B9-like |
|  | VITVI19G00177 | XP_002282717 | UDP-glycosyltransferase 79B9 |
|  | VITVI19G00564 | XP_010644495 | GDSL esterase/lipase At4g26790 |
|  | VITVI19G00735 | XP_002267870 | probable LRR receptor-like serine/threonine-protein kinase At4g26540 |
|  | VITVI19G00768 | XP_002275829 | probable galactinol--sucrose galactosyltransferase 1 |
|  | VITVI19G01555 | XP_002266113 | PREDICTED: uncharacterized protein LOC100254368 isoform X2 |
|  | VITVI19G02057 | XP_010644553 | zinc transporter 11 |
|  | VITVI19G02058 | XP_010644555 | PREDICTED: uncharacterized protein LOC104877619 |
|  | VITVI19G02100 | XP_010644663 | heavy metal-associated isoprenylated plant protein 47 |
| Group D | VITVI00G04671 | CBI18830 | unnamed protein product, partial |
|  | VITVI01G01476 | XP_002274353 | defensin Ec-AMP-D2 |
|  | VITVI01G01644 | XP_002271641 | cytochrome P450 78A4 |
|  | VITVI01G01884 | XP_010648750 | lectin |
|  | VITVI02G00287 | XP_002270961 | WAT1-related protein At3g30340 isoform X2 |
|  | VITVI02G00395 | XP_010661260 | probable (S)-N-methylcoclaurine 3'-hydroxylase isozyme 2 |
|  | VITVI03G00407 | XP_010647827 | cytochrome P450 CYP82D47 |
|  | VITVI03G00413 | XP_002281431 | cytochrome P450 82C4 |
|  | VITVI03G04145 | XP_002281361 | cytochrome P450 82C4 |
|  | VITVI03G04204 | RVW61427 | hypothetical protein CK203_031980 |
|  | VITVI04G01519 | XP_002278547 | F-box protein SKIP23-like |
|  | VITVI05G00009 | RVW99068 | UDP-glucuronic acid decarboxylase 2 |
|  | VITVI05G00018 | RVW99169 | Methylecgonone reductase |
|  | VITVI05G00299 | XP_019075476 | nuclear transcription factor Y subunit B-5-like |
|  | VITVI05G01940 | XP_010650076 | early nodulin-75 isoform X1 |
|  | VITVI05G01943 | XP_010650075 | early nodulin-75-like |
|  | VITVI06G00440 | XP_002281193 | osmotin-like protein |
|  | VITVI08G00756 | XP_010653398 | PREDICTED: uncharacterized protein LOC104880017 |
|  | VITVI08G01172 | XP_002262680 | cation/H(+) antiporter 20 isoform X1 |
|  | VITVI10G01384 | RVW78728 | Chaperone protein dnaJ C76, chloroplastic |
|  | VITVI11G00339 | RVW47516 | Heat stress transcription factor C-1 |
|  | VITVI11G00497 | XP_002285354 | auxin-responsive protein IAA9 isoform X1 |
|  | VITVI11G01506 | XP_002277450 | PREDICTED: uncharacterized protein LOC100255413 |
|  | VITVI12G02718 | XP_002263256 | protein TIFY 10A |
|  | VITVI14G00149 | XP_002279031 | bidirectional sugar transporter SWEET16 |
|  | VITVI14G01783 | XP_002274582 | bidirectional sugar transporter SWEET6b |
|  | VITVI14G04262 | XP_010660228 | myb-related protein Myb4 |
|  | VITVI16G01016 | XP_002267897 | 25.3 kDa vesicle transport protein isoform X1 |
|  | VITVI16G01978 | XP_002266583 | acidic endochitinase |
|  | VITVI18G00122 | XP_034676159 | uncharacterized protein LOC117906969 |
|  | VITVI18G00730 | XP_002285006 | probable isoaspartyl peptidase/L-asparaginase 2 |
|  | VITVI19G01748 | XP_002266025 | organic cation/carnitine transporter 3-like |
